# Daily Gene Expression Rhythms in Rat White Adipose Tissue Do Not Differ Between Subcutaneous and Intra-Abdominal Depots

**DOI:** 10.3389/fendo.2018.00206

**Published:** 2018-04-30

**Authors:** Rianne van der Spek, Eric Fliers, Susanne E. la Fleur, Andries Kalsbeek

**Affiliations:** ^1^Department of Endocrinology and Metabolism, Academic Medical Center (AMC), University of Amsterdam, Amsterdam, Netherlands; ^2^Hypothalamic Integration Mechanisms, Netherlands Institute for Neuroscience (NIN), Amsterdam, Netherlands

**Keywords:** circwave, visceral WAT, retroperitoneal WAT, lipid metabolism, circadian

## Abstract

White adipose tissue (WAT) is present in different depots throughout the body. Although all depots are exposed to systemic humoral signals, they are not functionally identical. Studies in clock gene knockout animals and in shift workers suggest that daily rhythmicity may play an important role in lipid metabolism. Differences in rhythmicity between fat depots might explain differences in depot function; therefore, we measured mRNA expression of clock genes and metabolic genes on a 3-h interval over a 24-h period in the subcutaneous inguinal depot and in the intra-abdominal perirenal, epididymal, and mesenteric depots of male Wistar rats. We analyzed rhythmicity using CircWave software. Additionally, we measured plasma concentrations of glucose, insulin, corticosterone, and leptin. The clock genes (*Bmal1/Per2/Cry1/Cry2/RevErb*α*/DBP*) showed robust daily gene expression rhythms, which did not vary between WAT depots. Metabolic gene expression rhythms (*SREBP1c/PPAR*α*/PPAR*γ*/FAS/LPL/Glut4/HSL/CPT1b/leptin/visfatin/resistin*) were more variable between depots. However, no distinct differences between intra-abdominal and subcutaneous rhythms were found. Concluding, specific fat depots are not associated with differences in clock gene expression rhythms and, therefore, do not provide a likely explanation for the differences in metabolic function between different fat depots.

## Introduction

Sustained disturbances in daily rhythmicity (e.g., shift work, jet lag) increase the risk to develop obesity and related metabolic disease (1). Storage in and release of lipids from white adipose tissue (WAT) are regulated processes that anticipate rest-activity and feeding cycles. WAT is abundantly present throughout the body in different fat depots. In male rats, the main depots are located underneath the skin in the inguinal area [subcutaneous white adipose tissue (sWAT)], and in the abdominal cavity (intra-abdominal depots): perirenal- (pWAT, retroperitoneal, next to the kidney), epididymal- (eWAT, connected to and lining the epididymis), and mesenteric WAT (mWAT, intraperitoneal, lining the gastrointestinal tract).

Interestingly, although all depots are exposed to systemic humoral signals, such as circulating hormones and nutrients, subcutaneous and intra-abdominal WAT depots are not functionally identical (2, 3). For example, retroperitoneal WAT is more responsive to metabolic challenges (fasting/refeeding) compared to subcutaneous WAT (4). Additionally, in various lipodystrophy syndromes subcutaneous fat stores are depleted, while simultaneously intra-abdominal WAT accumulates (5), pointing to differential differentiation and proliferation of adipose depots. Moreover, excess storage of intra-abdominal WAT is associated with adverse health effects, whereas subcutaneous WAT accumulation might be beneficial (6–9). Moreover, effects of sex hormones (10) and glucocorticoid treatment differ between WAT depots (11). To date, it is unexplained where these differences originate and how they are integrated to ensure that the net effect of the WAT depots results in energy homeostasis.

Like most peripheral tissues, WAT depots encompass an intrinsic molecular clockwork based on a transcriptional–translational feedback loop. Since clock proteins regulate the expression of genes involved in many (metabolic) processes within a cell, clock rhythms play an important role in tissue function. The core loop of the molecular clock is formed by the Clock:Bmal1 heterodimer that upregulates expression of the Period 1–3 (Per 1–3) and Cryptochrome 1–2 (Cry1–2) proteins. Per’s and Cry’s subsequently heterodimerize, translocate to the nucleus, and inhibit Clock:Bmal1 activity. As a consequence, Clock:Bmal1 transcriptional activity drops, which reduces the transcription of *Per* and *Cry* genes, thereby activating Clock:Bmal1 again. The retinoic acid-related orphan nuclear receptors, RevErb and ROR, represent additional regulatory loops that enhance the robustness of the core loop, by binding to retinoic acid-related orphan receptor response elements on the Bmal1 promotor (12).

Studies in clock gene knockout animals and studies in shift workers suggest daily rhythms play an important role in lipid metabolism. For example, the arrhythmic CLOCKΔ19 C57BL/6J mouse is hyperglycemic, hyperlipidaemic, hyperleptinaemic, and hypoinsulinaemic, with increased body weight and visceral adiposity (13, 14). Moreover, disruption of the adipocyte clock by adipose tissue specific deletion of Bmal1, results in obesity, temporal changes in plasma concentration of fatty acids, and altered hypothalamic appetite regulation (15). In CLOCKΔ19 C57BL/6J mice, the impaired adipose tissue clock may directly affect diurnal transcriptional regulation of lipid homeostasis, reducing FFA/glycerol mobilization from WAT stores (16).

To determine whether differences in daily rhythmicity between WAT depots could explain differences in depot function, we analyzed rhythmicity of clock gene (*Bmal1, Per2, Cry1, Cry2, RevErb*α, and *DBP*) and metabolic gene expression (*SREBP1c, PPAR*α, *PPAR*γ, *FAS, LPL, Glut4, HSL, CPT1b, leptin, visfatin*, and *resistin*) in different intra-abdominal and subcutaneous WAT depots. We conclude that differences in the molecular clock or clock-controlled genes do not provide a major explanation for the differences in metabolic function between the different fat depots. Furthermore, our results suggest that in *ad libitum* feeding conditions the timing of subcutaneous WAT clock gene rhythms can be extrapolated to those of intra-abdominal WAT depots.

## Results

### Overall Rhythmicity of Gene Expression in Adipose Tissue

**Figure 1:**
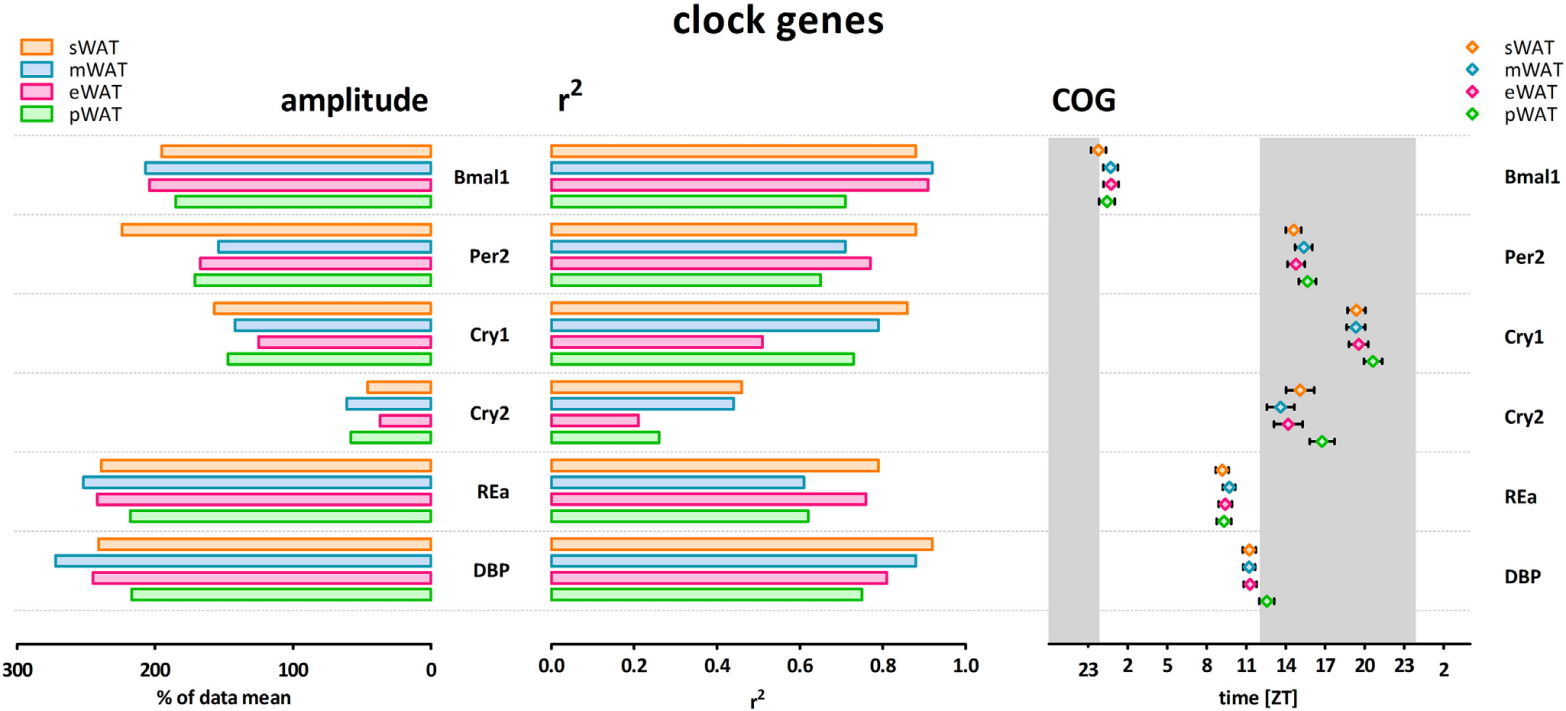
Clock genes show high amplitude, high *r*^2^ (low inter-individual variability) and low variability in center of gravity (COG) in subcutaneous and intra-abdominal white adipose tissue (WAT) depots. sWAT, subcutaneous; mWAT, mesenteric; eWAT, epididymal; pWAT, perirenal. Gray bars indicate the dark phase (ZT 12–24). Individual expression curves for each gene and WAT depot can be found in Figure S1 in Supplementary Material.

**Figure 2:**
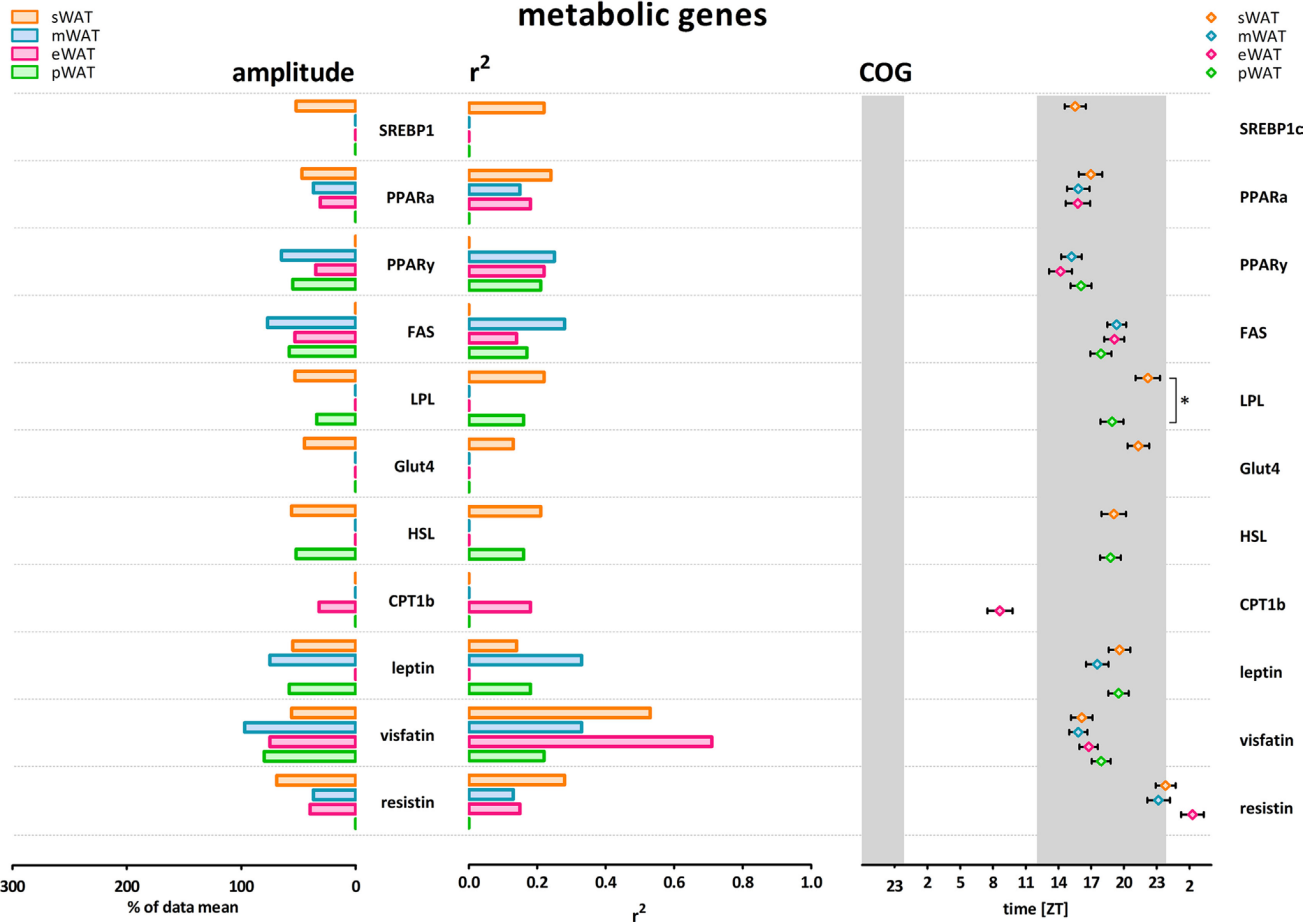
Metabolic genes show modest amplitude, modest *r*^2^ (inter-individual variability) and higher variability in center of gravity (COG) in subcutaneous and intra-abdominal white adipose tissue (WAT) depots. LPL peaked significantly earlier in pWAT compared to sWAT (two-tailed *t*-test *F* = 1,133; *p* = 0.0342; difference = 3.2 ± 1.5 h) sWAT; subcutaneous, mWAT; mesenteric, eWAT; epididymal, pWAT; perirenal. Gray bars indicate the dark phase (ZT 12–24). Absence of amplitude, *R*^2^, and COG values indicates absence of significant rhythmicity, not absence of gene expression. Individual expression curves for each gene and WAT depot can be found in Figure S1 in Supplementary Material.

**Figure 3:**
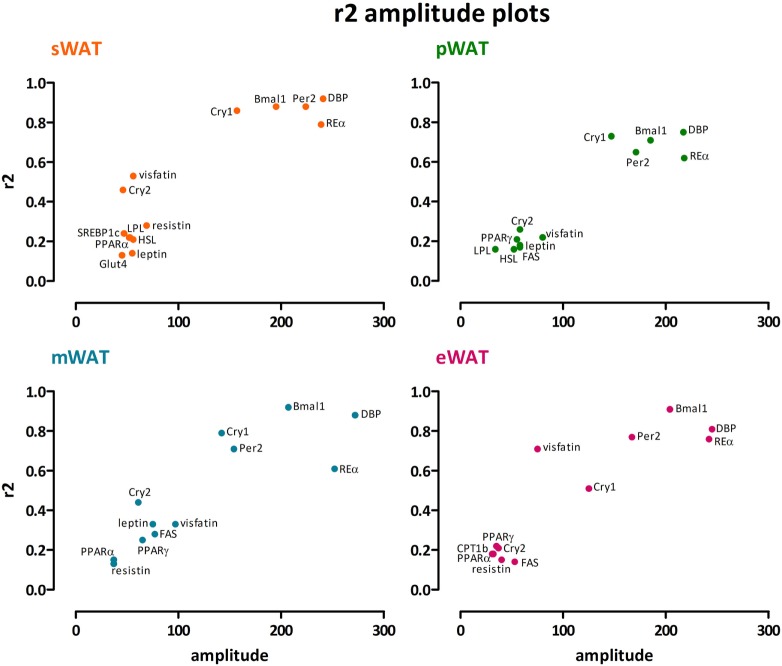
Clock genes show high amplitude together with high *r*^2^, whereas for metabolic genes modest amplitudes go along with low *r*^2^ values. A clear exception is Cry2, which showed much lower amplitude and *r*^2^ values than the other clock genes. We found no distinct differences between subcutaneous and intra-abdominal white adipose tissue (WAT) depots. sWAT, subcutaneous; mWAT, mesenteric; eWAT, epididymal; pWAT, perirenal. Individual expression curves for each gene and WAT depot can be found in Figure S1 in Supplementary Material.

To describe rhythmicity, we considered the following factors to be important; peak time [expressed as center of gravity; COG (see Materials and Methods)], robustness, and amplitude. Therefore, we analyzed variation between depots for these factors. We defined “robustness” of a rhythm as: uniformity between cycles and/or animals measured by three characteristics; period, phase, and shape of wave. *R*^2^ values indicate goodness of fit on a scale from 0 to 1, i.e., how well the Circwave curve describes the data. Thus, *r*^2^ values close to 1 indicate that individual samples deviate very little from the curve and, therefore, show little inter-animal variation in period, phase and shape of wave, and can be called robust. Clock gene and metabolic gene expression per WAT depot, *r*^2^ (inter-individual variability) and amplitude are plotted for each gene in Figures 1 and 2. For all WAT depots, clock gene expression was highly rhythmic, with large amplitudes (range 125–272) and low variability (*r*^2^ range 0.61–0.92) between animals. A clear exception was *Cry 2*, which showed much lower amplitude (range 37–61) and *r*^2^ values (range 0.21–0.46) than the other five clock genes investigated. Metabolic genes on the other hand exhibited weak rhythmicity with lower amplitude (range 0–97) and high variability (*r*^2^ range 0.21–0.71) between animals (Figures 2 and 3), similar to or lower than the values for *Cry 2*. Individual expression curves for each gene and WAT depot can be found in Figure S1 in Supplementary Material.

### Clock Gene Expression Comparison Between WAT Depots

Clock gene expression showed pronounced daily rhythms in all WAT depots. *R*^2^ values showed little variation between depots, and limited variation between genes (Figure 1). *Cry2* showed the most pronounced variation between WAT depots; *r*^2^ values for pWAT (0.26) and eWAT (0.21) were about 50% smaller than for sWAT (0.46) and mWAT (0.44). Amplitude variations were limited between WAT depots (Figures 1 and 3). Of note, for most clock genes the lowest amplitude was found in pWAT. For *Per2* mRNA the amplitude in sWAT was clearly higher compared to the other depots. Peak time for the different clock gene curves (depicted as COG) was very similar between WAT depots (Figure 1, one-way ANOVA: ns). *Bmal1* peaked in the beginning of the light phase (ZT24) and as expected, *Per* and *Cry* rhythms were in antiphase, to *Bmal1*. *Per2* (ZT15–16), and *Cry2* (ZT14–17) peaked in the early dark period, whereas *Cry 1* (ZT19–20) mRNA peaked in the middle of the dark period. *RevERB*α (ZT9–10) and *DBP* (ZT11–12) mRNA were high at the end of the light phase (Figure 1).

#### Metabolic Gene Expression Comparison Between WAT Depots

Daily rhythms in metabolic gene expression were present; however, rhythmicity was not as robust (higher variability and lower amplitudes) as it was for clock genes (Figure 2). Rhythmicity was not apparent for every gene and for some genes not in every WAT depot. Absence of amplitude, *R*^2^, and COG values in Figure 2 indicates absence of significant rhythmicity, not absence of gene expression (see Figure S1 in Supplementary Material for individual gene expression curves). *R*^2^ values were modest overall; *r*^2^ was highest for *visfatin* in eWAT and sWAT (Figures 2 and 3). Similarly, amplitudes in metabolic genes were modest overall, i.e., <100%. Peak time (COG) for most metabolic genes did not differ between WAT depots. However, for *LPL* significant differences were detected (Figure 2). *LPL* peaked significantly earlier in pWAT compared to sWAT (two-tailed *t*-test *F* = 1,133; *p* = 0.0342; difference = 3.2 ± 1.5 h).

### Daily Rhythms in Plasma Hormone and Substrate Levels

**Figure 4:**
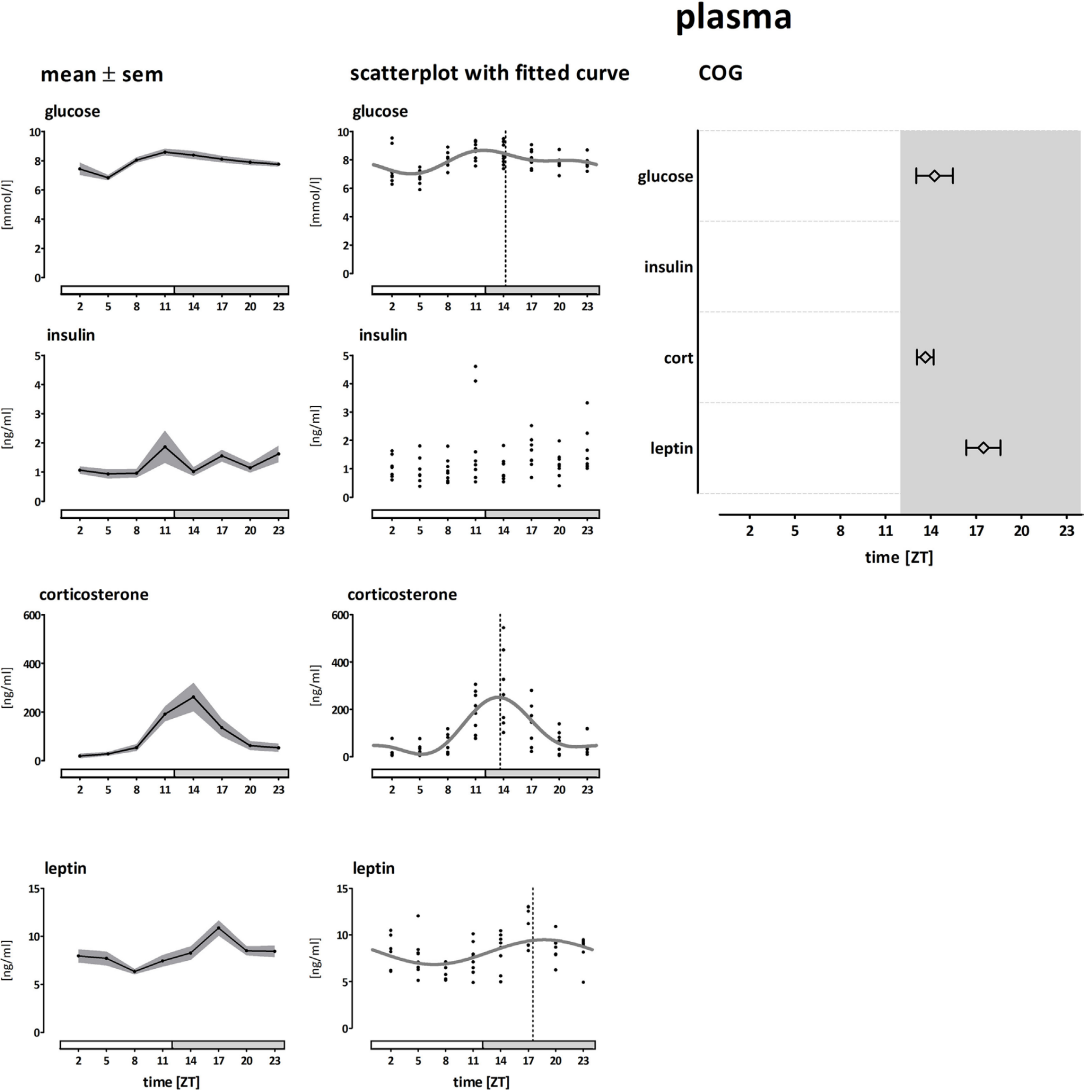
Plasma concentrations of glucose, insulin, corticosterone, and leptin. Left panel shows the mean (±SEM) plasma concentrations. The middle panel shows the individual data points and Circwave curves, the dotted line indicates the Center of Gravity (COG). The right panel shows the COG (±SD). Absence of the Circwave curve and the COG line indicates absence of significant rhythmicity as analyzed by Circwave. Gray bars indicate the dark phase (ZT 12–24).

Plasma levels and COGs of glucose, insulin, corticosterone, and leptin are shown in Figure 4. Plasma glucose concentrations were modestly rhythmic and peaked at the transition from light to dark phase (~ZT14, amplitude 21%, ANOVA: *F* = 4.78; *p* < 0.001, CIRCWAVE: *r*^2^ = 0.35; *p* < 0.001). Plasma insulin concentrations were not rhythmic, but showed a greater variation at the end of the light phase (ANOVA: *F* = 1,867; *p* = 0.0923). Plasma corticosterone concentrations were highly rhythmic and peaked slightly before the glucose peak (~ZT13, amplitude 237%, ANOVA: *F* = 8,852; *p* < 0.001, CIRCWAVE: *r*^2^ = 0.53; *p* < 0.001). Plasma leptin concentrations were modestly rhythmic and peaked in the middle of the dark phase (~ZT17, amplitude 32%, ANOVA: *F* = 4,073; *p* < 0.005, CIRCWAVE: *r*^2^ = 0.22; *p* < 0.001).

## Discussion

Different WAT depots have different functions, and increase and reduce their mass differentially, as illustrated by several metabolic disorders that result in loss of mainly subcutaneous or gain of mainly intra-abdominal (visceral) fat mass. Rhythmicity plays an important role in lipid metabolism, and clock gene rhythms have been described for some but not all WAT depots in rodents (17–20) and in humans (21, 22). We, therefore, hypothesized that differences in rhythmicity might explain differences in depot function and analyzed rhythmicity of gene expression in subcutaneous and different intra-abdominal WAT depots. However, in contrast to our hypothesis, we did not observe clear differences in clock gene rhythms between different WAT depots (Figures 1–3). Moreover, most metabolic genes only showed modest or non-significant rhythmicity. Therefore, differences in the molecular clock or clock-controlled genes do not provide a major explanation for the differences in metabolic function between the different fat depots.

We observed robust rhythms in clock gene expression in all four fat depots studied (*Bmal1, Per2, Cry1, Cry2, RevErb*α, *DBP*), with a peak time that was similar to what has been described previously for Wistar rats (20, 23, 24) and other rodent species (17–19). Only few studies have measured clock gene expression rhythms in both epididymal WAT and subcutaneous (inguinal) WAT; one found lower amplitudes in subcutaneous WAT compared to epididymal (19), whereas in the other study, amplitudes were marginally smaller in epididymal WAT compared to subcutaneous WAT (18). In our data set, amplitude, robustness, or timing (COG) were not significantly different between mesenteric-, perirenal-, epididymal-, and subcutaneous WAT depots. This is the first study to extensively compare clock gene rhythms in subcutaneous and different abdominal WAT depots simultaneously. Because we did not observe pronounced differences between depots under these untreated, *ad libitum* feeding conditions, this suggests that with regard to clock gene expression rhythms the results from subcutaneous inguinal WAT—which in humans is far less invasive to biopsy compared to internal WAT depots—may be extrapolated to other depots.

In contrast to the overt day/night rhythms in clock gene expression, expression of metabolic genes showed no profound rhythmicity. Metabolic genes that did show significant rhythmicity mostly showed peak expression in the active (dark) phase. These findings are in line with data from mice (25). Metabolic genes are influenced by multiple circulating factors, such as corticosterone, insulin and nutrients, either directly (e.g., *via* a glucocorticoid response element) or *via* transcription factors (e.g., *SREBP1c, PPARs*) (26–28).

The daily rhythms in plasma corticosterone and glucose are independent of the daily rhythm in feeding behavior, whereas plasma levels of insulin and glucagon are mainly regulated by food intake (29, 30). Corresponding with previous data, we found that plasma concentrations of corticosterone and glucose peaked at the onset of the active phase. *PPAR*α and *-*γ are glucocorticoid sensitive transcription factors (31), and indeed for *PPARs* we observed an expression peak with a similar timing as that of plasma corticosterone. Plasma insulin concentrations did not show a significant day/night rhythm, but rather followed feeding activity with three spikes during the dark phase. Several genes encoding for proteins involved with energy storage in the fed state (*SREBP1c, PPAR*γ, *LPL, FAS, Glut4, leptin, resistin*) showed a spiky expression pattern similar to the insulin curve (Figure S1 in Supplementary Material). These genes are likely influenced by feeding-induced insulin release, or by nutrients directly (e.g., *via* PPRE) (28).

LPL serves as a gatekeeper that controls local fatty acid uptake into cells by catalyzing the hydrolysis of circulating triglycerides. Transcription of *LPL* is upregulated by fatty acids, SREBP1c and PPARγ and downregulated and inactivated in the fasted state by glucocorticoids, catecholamines, and decreased levels of PPARγ and SREBP1c. In our data, *LPL* showed a 3-h delayed expression in sWAT compared to pWAT. In line with upregulation during the feeding period, we observed peak expression in eWAT when animals are eating. The delayed peak in *LPL* expression in sWAT fits with the hypothesis that intra-abdominal WAT is primarily functional in short term metabolic regulation, and sWAT takes up the lipid overflow for long term energy storage (2). LPL protein concentration also peaks in the active period, but it remains to be determined how the rhythms in mRNA and protein content correspond to activity levels, as most physiological variation in LPL activity appears to be driven by posttranslational mechanisms by extracellular proteins (32).

Leptin concentrations peaked in the middle of the active (dark) phase (Figure 4), in line with previous experiments (33). This peak in plasma corresponds with the rhythm in *leptin* mRNA in fat tissue (Figure S1 in Supplementary Material Leptin). We observed the clearest correlation between plasma leptin concentrations and *leptin* mRNA expression in mWAT (Figure S2 in Supplementary Material Leptin correlation). Although we cannot compare absolute mRNA expression levels between depots (due to the number of samples we had to analyze each depot as a separate batch), others have shown that *leptin* mRNA levels are generally much higher in intra-abdominal depots, compared to subcutaneous depots (34). Furthermore, they found plasma leptin levels correlated only with *leptin* expression in mWAT, but not any of the other WAT depots (34), which is in line with the correlations we observed between *leptin* mRNA and plasma leptin concentrations. Another study comparing *leptin* mRNA expression rhythms between WAT depots in rats found expression curves quite similar to our data in mesenteric and perirenal (retroperitoneal) WAT. However, they found epididymal WAT to be rhythmic, in contrast to our dataset. These different observations accentuate the modest amplitude of the leptin expression rhythms; hence conclusions should be drawn with caution. In contrast to rodents, in humans subcutaneous fat tissue is the primary source of circulating leptin levels (35, 36). Therefore, the contribution from subcutaneous leptin mRNA to both plasma leptin levels and their rhythm would be expected to be more important in humans. Indeed *leptin* mRNA is rhythmic in human subcutaneous tissue as well (21). One explanation for this discrepancy between rodents and humans could be a different ratio of subcutaneous versus intra-abdominal fat mass.

A number of other factors in our study may have contributed to variation in gene expression, of both clock and metabolic genes. First, our animals had *ad libitum* access to food, which could have induced small variations in timing of food intake between animals which might have led to less robust rhythms. Second, we have used Circwave to analyze rhythmicity in our data. Circwave recognizes wave forms using Fourier transformation whereby harmonics are added in a step-wise regression like fashion (using *F*-testing). This method is based on the assumption that the rhythms consist of one or more sine waves, and that noise variance is Gaussian (normally) distributed and independent of measurement magnitude. Therefore, it limits the recognition of spiky and saw tooth-shaped wave forms (37). Although the choice for this method might influence the sensitivity with which we were able to recognize rhythms, it will only affect our main conclusion (no rhythmic differences between depots) if there would be major differences in shape of wave between the WAT depots. Looking at the raw data sets (Figure S1 in Supplementary Material), we may underestimate spiky rhythmicity of insulin or nutrient regulated genes. Nevertheless, alternative methods do not allow for estimation of amplitudes and phases (37), which were our main outcome measures.

We found no evidence that differences in rhythmicity in clock or metabolic genes underlie the functional differences described for the different WAT depots. Alternative explanations for functional differences are differences in pre-adipocyte lineage (2), differences in innervation, or differences in local regulation. Typically, the hypothalamus integrates peripheral signals and ensures energy homeostasis by regulating peripheral energy metabolism *via* humoral pathways and the autonomic nervous system (ANS). Indeed, intra-abdominal and subcutaneous WAT are innervated by separate sets of neurons (38), all the way up to the pre-autonomic neurons in the hypothalamus (39). Subcutaneous (inguinal) WAT gains more adipose cells after denervation compared to intra-abdominal (retroperitoneal) WAT (40). These data indicate that differential innervation can contribute to functional differences between WAT depots, but apparently do not result in differences in rhythmicity. Whether differences in functionality are indeed depending on differences in autonomic activity at the level of WAT still needs to be proven. Moreover, it could well be that ANS mediated differences in WAT functionality only surface during positive or negative energy balance.

Concluding, in contrast to our hypothesis, we did not observe clear differences in (clock) gene expression rhythms between different WAT depots. Moreover, we found only modest rhythmicity in metabolic gene expression rhythms, and no results that could explain differences in metabolic function between the different WAT depots. Therefore, functional differences between WAT depots likely stem from other regulatory levels (i.e., translational) or pathways.

## Materials and Methods

### Animals

Sixty-four male Wistar rats (Harlan, Horst, Netherlands) were kept on a 12/12-h light/dark cycle (lights on at 0700 hours), at a room temperature (20 ± 2°C), with four to six animals per cage. Thirty-two animals were housed in a room with a reversed light/dark cycle. The experiment was carried out in October. After arrival, animals were allowed to adapt to their new environment and the lighting schedule for 3 weeks before the experiment. Food and water were provided *ad libitum*. The experiment was conducted under approval of the Local Animal Welfare Committee.

### Experiment

To obtain WAT tissues and plasma, animals were anesthetized with isoflurane and killed by decapitation at a 3-h interval starting at ZT2 (ZT14 for reversed light–dark cycle) and ending at ZT11 (ZT23 for reversed light–dark cycle). At every time point, four animals were obtained from both rooms, thereby spreading the total sampling period over a 48-h period.

Intra-abdominal perirenal (pWAT), epididymal (eWAT), and subcutaneous inguinal (sWAT) white adipose tissues were dissected and snap frozen in liquid nitrogen. Intra-abdominal mesenteric (m)WAT was separated from the gastrointestinal tract and pancreas and snap frozen in liquid nitrogen. Blood was collected in heparinized tubes.

### Plasma Analyses

Following decapitation trunk blood was collected and kept on ice in heparinized tubes until centrifugation for 15 min at 3,000 rpm at 4°C. Plasma was transferred to a clean tube and stored at −20°C until use. Plasma glucose was measured using a Biosen apparatus (EKF diagnostics, Cardiff, UK). Plasma insulin, leptin, and corticosterone were measured using a radio immuno assay (Merck Millipore, Billerica, MA, USA).

### Gene Expression Analysis

#### RNA Extraction

Total RNA (tRNA) was extracted from approximately 100 mg of adipose tissue, using the RNeasy lipid kit (Qiagen Benelux, Venlo, Netherlands), with on-column DNAse treatment using RNAse-free DNAse (Qiagen Benelux, Venlo, Netherlands), according to the manufacturer’s protocol. tRNA was measured on a Nanodrop 1000 (Thermo Fisher Scientific, Waltham, MA, USA) and diluted to equal concentrations.

#### cDNA Synthesis

cDNA was synthesized with the Transcriptor First Strand cDNA synthesis kit from Roche (Roche, Almere, Netherlands) using anchored oligo (dT)18 primers and 18 ng tRNA per microliter cDNA. To check for genomic DNA contamination in the extracted RNA, we included several samples for which we replaced reverse transcriptase with PCR grade water (−RT controls). If the fluorescence curve of one of the −RT controls lay within 10 cycles of the cDNA sample with the lowest expression, the PCR assay was rejected because of potential genomic DNA contamination.

#### RT-qPCR

Gene expression was analyzed by real-time RT-qPCR on a LightCycler 480 system (Roche, Almere, Netherlands), using SybrGreen I Master, primer pairs, PCR grade water and cDNA. All primer pairs were designed intron-spanning if possible, and amplicon size and specificity was checked on electrophoresis gel. If the amplicon size matched and a single band was found, the PCR product was purified using a QIAquick PCR purification kit (Qiagen Benelux, Venlo, Netherlands). The purified PCR product was diluted and used in subsequent PCRs as a positive control combined with melting peak analysis.

#### LinRegPCR

For each PCR assay, PCR efficiency was checked for all samples individually using LinRegPCR. LinRegPCR software determines baseline fluorescence sets a Window-of-Linearity to calculate PCR efficiency. The starting RNA concentration expressed in arbitrary fluorescence units, is calculated using the mean PCR efficiency per sample, the Cq value per sample and the fluorescence threshold used to determine the Cq (41, 42). Samples that differed more than 0.05 from the efficiency median value were excluded from further analysis.

#### Normalization

To control for variation in the amount of mRNA input, gene expression levels of the target sequence were normalized to the expression of an endogenous control, hypoxanthine phosphoribosyl transferase (HPRT) gene expression (43).

Several commonly used reference genes show a circadian rhythm in their expression profile (44), and these rhythms may vary between tissues, species, and strains (45). HPRT was chosen as a reference gene because it expressed no, or only very low amplitude rhythms in our samples (data not shown). Additionally, all PCR data are expressed relative to ZT2, to allow comparison between WAT depots.

#### Genes of Interest

Primer sequences of clock genes *Bmal1, Per2, Cry1*, and *Cry2, RevErb*α and *DBP*, and metabolic genes *SREBP1c, PPAR*α, *PPAR*γ, *FAS, LPL, HSL, CPT1b, Glut4, leptin, visfatin*, and *resistin* have been published previously (27).

### Data Analysis and Statistics

For identification of outliers, we used Dixon’s *Q* test with two-tailed *Q*-values (46). Samples that were determined outliers were excluded from further analysis (Table S1 in Supplementary Material).

All data (plasma and PCR) are presented as mean ± SEM unless otherwise stated. *p* Values below 0.05 were considered statistically significant.

Variations between time points within one gene in one depot were evaluated by one-way ANOVA and rhythmicity was assessed using Circwave v1.4 (www.hutlab.nl). Circwave software fits one or more fundamental sinusoidal curves through the individual data points and compares this with a horizontal line through the data mean (a constant). If the fitted curve differs significantly from the horizontal line, the data set is considered rhythmic. Circwave provides the following information: number of sines in the fitted curve; data mean, the average of all data points with SD; Centre of Gravity (CoG), representing the general phase of the curve with SD; ANOVA *F* stat, *p*-value and *r*^2^; Circwave *F* stat, *p*-value and *r*^2^. Centre of Gravity (COG) SDs were calculated without assuming the data was circular, as rhythmicity of gene expression was one of the outcome measures.

Centre of gravity data per gene were compared between WAT depots using unpaired two-tailed *t*-test with *F* test. Variances did not differ between WAT depots.

Amplitudes of Circwave curves were calculated as percentages of data mean to enable comparison of amplitudes between data sets [difference between the zenith (highest point) and nadir (lowest point) and divided by the data mean (max − min/mean * 100%)].

## Ethics Statement

All the studies were approved by and performed according to the regulations of the Committee for Animal Experimentation of the Netherlands Institute for Neuroscience (NIN) of the Royal Netherlands Academy of Arts and Sciences (KNAW), Netherlands.

## Author Contributions

RS, EF, SF, and AK conceived and designed the experiments and wrote the paper. RS, SF, and AK performed the experiments and analyzed the data.

## Conflict of Interest Statement

The authors declare that the research was conducted in the absence of any commercial or financial relationships that could be construed as a potential conflict of interest.

## Supplementary Material

Figure S1 Individual expression curves for each gene and white adipose tissue depot.

Figure S2 Correlation between plasma leptin concentrations and leptin mRNA expression in mesenteric WAT.

Table S1 The number of samples after exclusion of outliers for the PCR results at each time point and for each gene investigated.

## Abbreviations

ANS, autonomic nervous system; COG, centre of gravity (see method section); WAT, white adipose tissue; ZT, zeitgeber time.
