# Supplementary material for: Daily Gene Expression Rhythms in Rat White Adipose Tissue Do Not Differ Between Subcutaneous and Intra-Abdominal Depots
Source: Front Endocrinol (Lausanne). 2018 Apr 30;9:206. doi: 10.3389/fendo.2018.00206 (PMC5936761; doi:10.3389/fendo.2018.00206)
Supplement: Figure S1 — Individual expression curves for each gene and white adipose tissue depot. [file image_1.PDF]

Bmal1

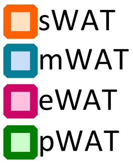

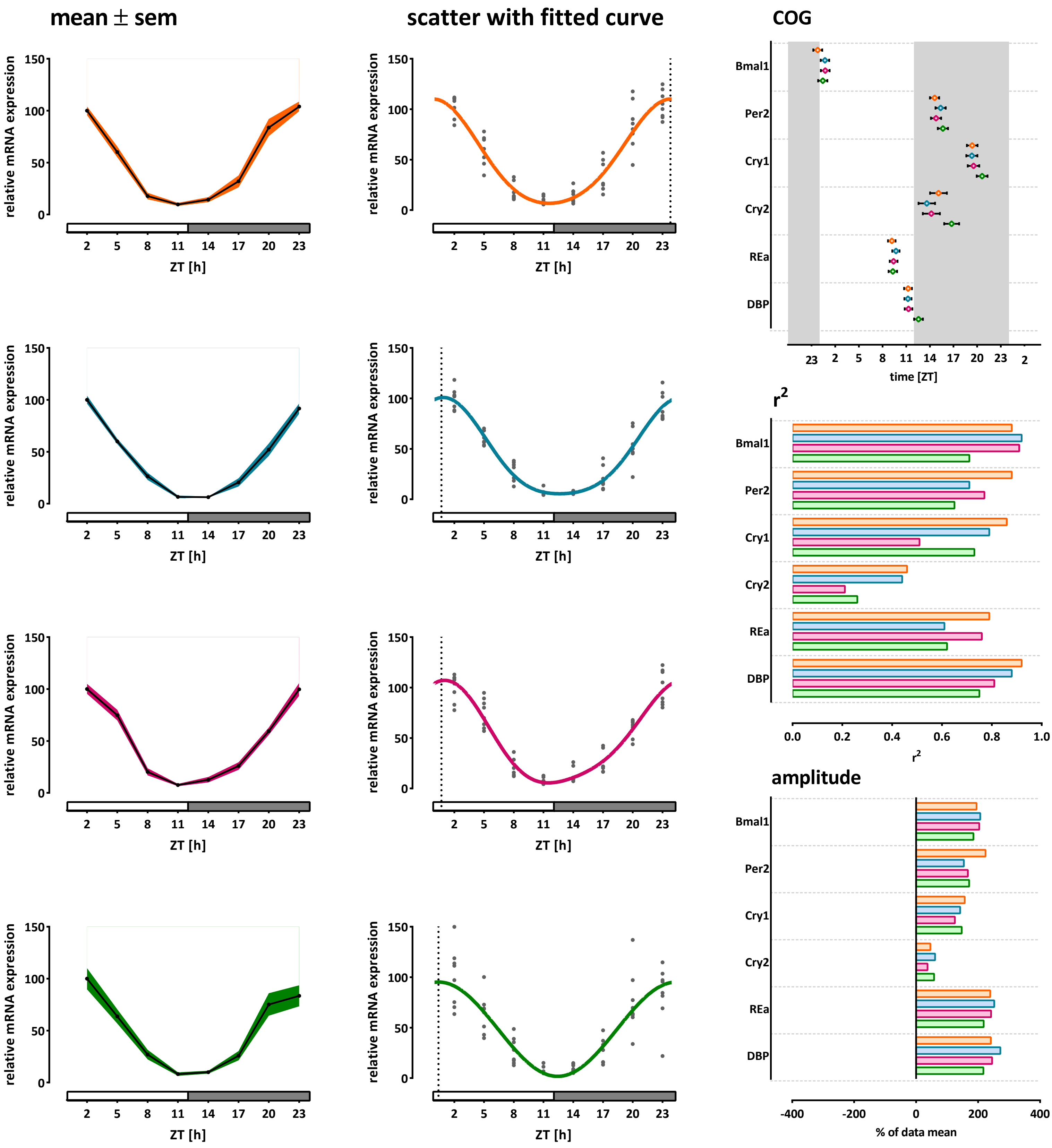

PCR data are shown in the left pannel (mean ± sem) and middle panel (scatter plot). All data are corrected for housekeeping gene expression and are expressed relative to ZT2 to allow for comparison between different PCR plates.

Circwave fitted curves are shown in the panel in the middle.
COG(Centre Of Gravity) is a general phase marker. Depicted in the upper right panel and by the dotted line in the scatterplots in the middle panel.
r^2 describes goodness of fit, shown in the right hand panel. 1.0 indicates perfect description of individual data points and thus little variation between animals.
amplitudes are expressed as max-min/data mean, shown in the lower right panel.

Per2

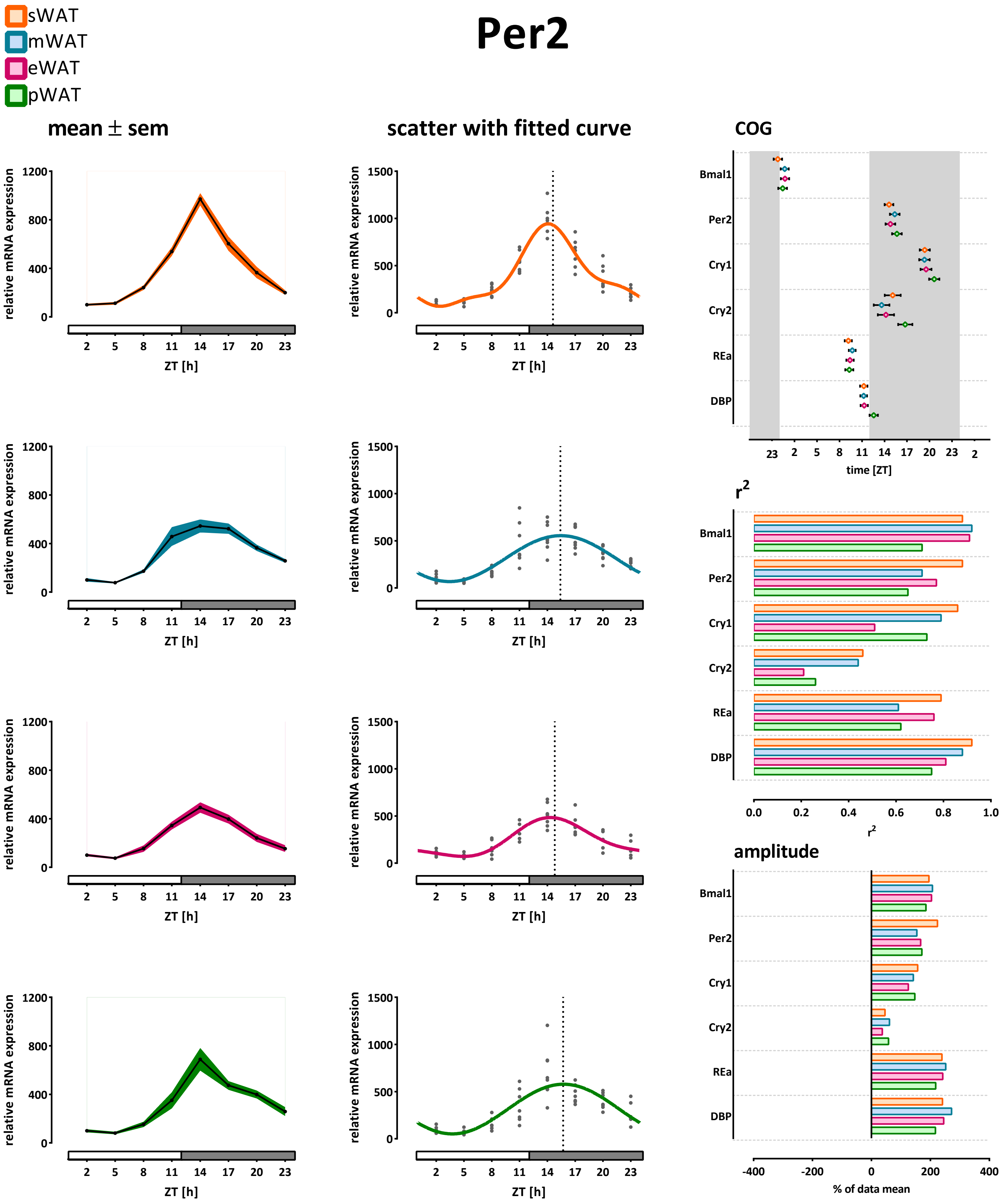

PCR data are shown in the left pannel (mean ± sem) and middle panel (scatter plot). All data are corrected for housekeeping gene expression and are expressed relative to ZT2 to allow for comparison between different PCR plates.

Circwave fitted curves are shown in the panel in the middle.
COG(Centre Of Gravity) is a general phase marker. Depicted in the upper right panel and by the dotted line in the scatterplots in the middle panel.
r^2 describes goodness of fit, shown in the right hand panel. 1.0 indicates perfect description of individual data points and thus little variation between animals.
amplitudes are expressed as max-min/data mean, shown in the lower right panel.

Cry1

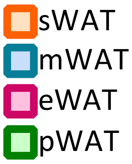

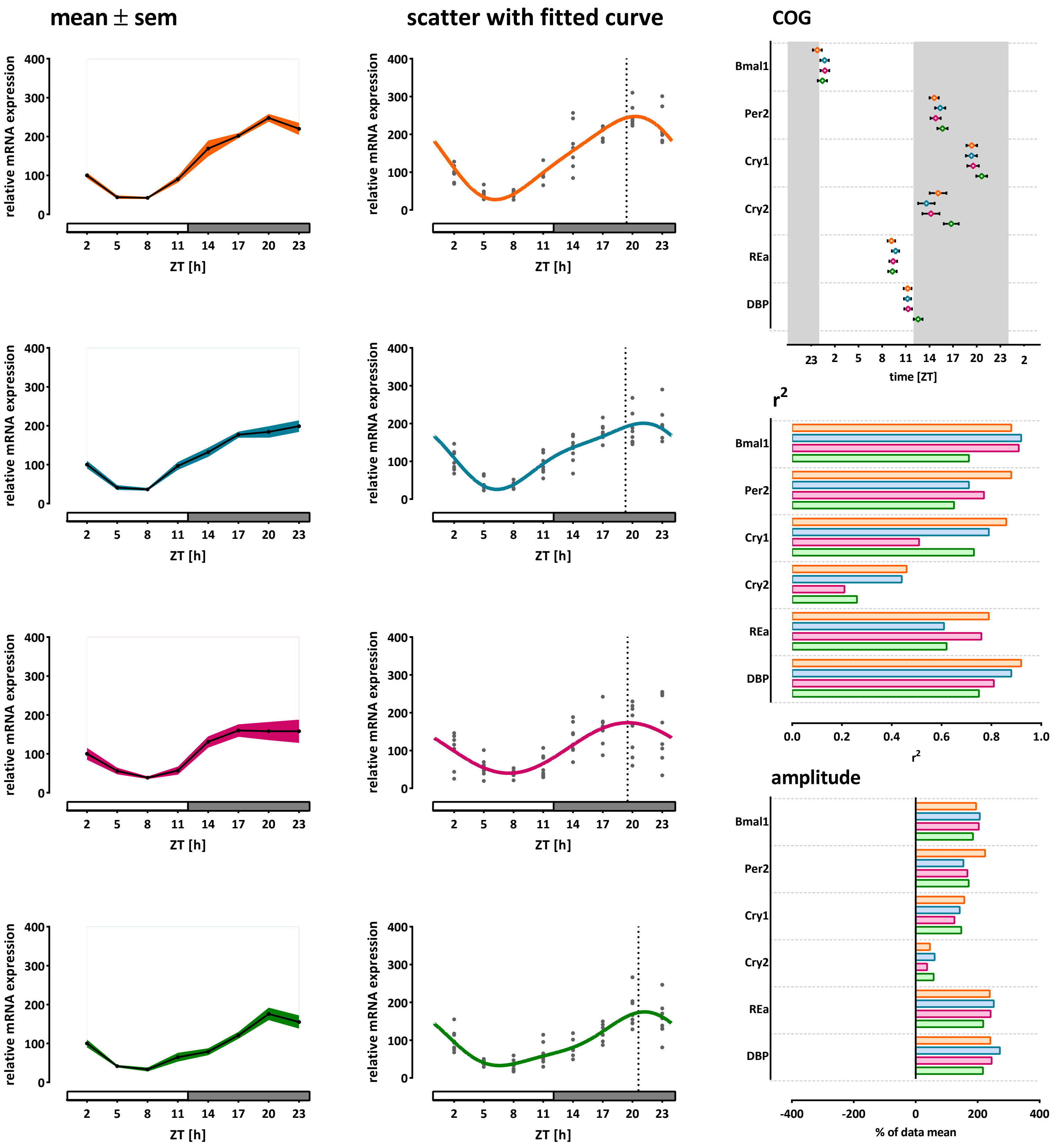

PCR data are shown in the left pannel (mean ± sem) and middle panel (scatter plot). All data are corrected for housekeeping gene expression and are expressed relative to ZT2 to allow for comparison between different PCR plates.

Circwave fitted curves are shown in the panel in the middle.
COG(Centre Of Gravity) is a general phase marker. Depicted in the upper right panel and by the dotted line in the scatterplots in the middle panel.
r^2 describes goodness of fit, shown in the right hand panel. 1.0 indicates perfect description of individual data points and thus little variation between animals.
amplitudes are expressed as max-min/data mean, shown in the lower right panel.

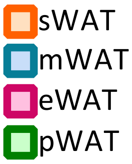

Cry2

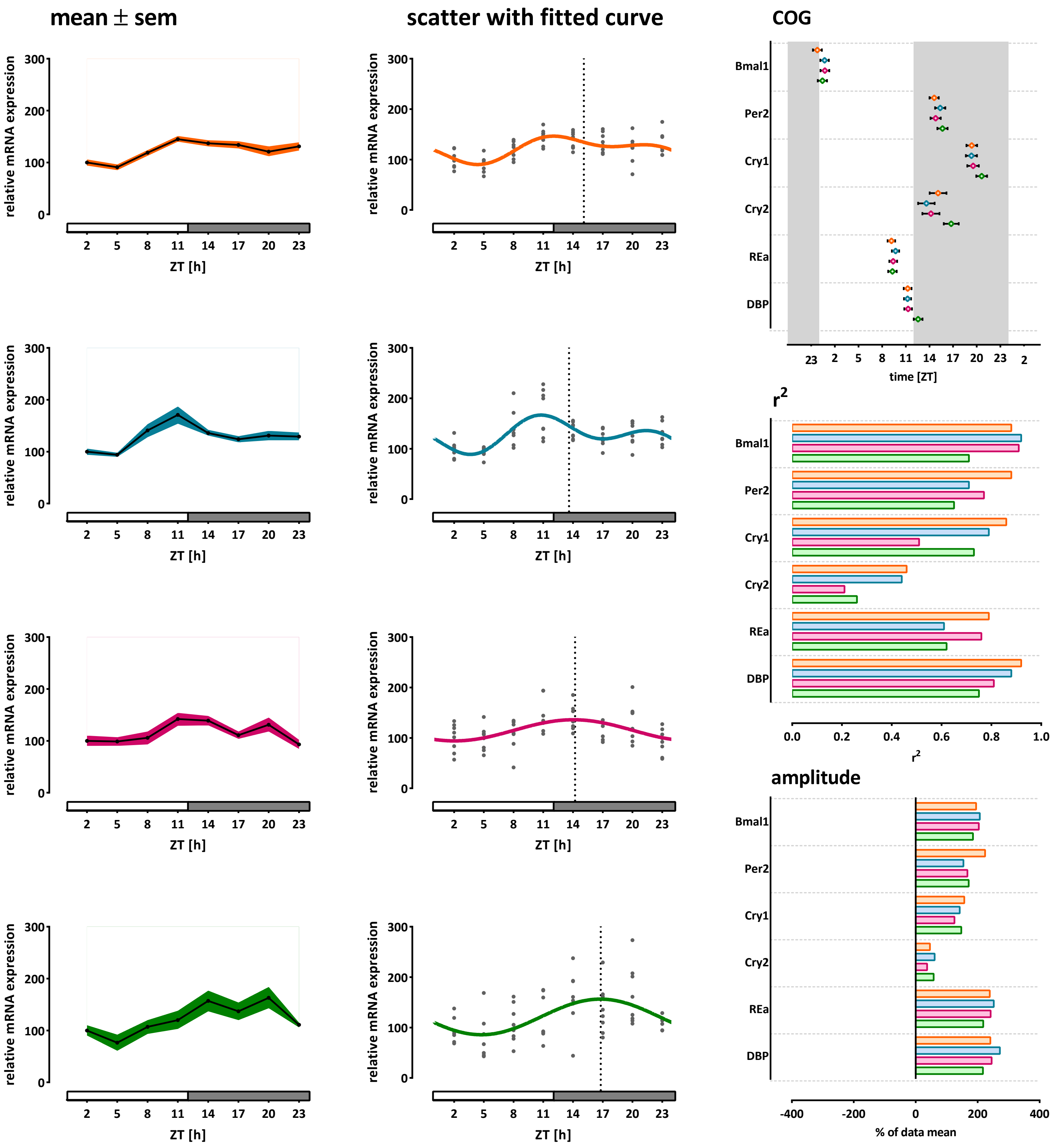

PCR data are shown in the left pannel (mean ± sem) and middle panel (scatter plot). All data are corrected for housekeeping gene expression and are expressed relative to ZT2 to allow for comparison between different PCR plates.

Circwave fitted curves are shown in the panel in the middle.
COG(Centre Of Gravity) is a general phase marker. Depicted in the upper right panel and by the dotted line in the scatterplots in the middle panel.
r^2 describes goodness of fit, shown in the right hand panel. 1.0 indicates perfect description of individual data points and thus little variation between animals.
amplitudes are expressed as max-min/data mean, shown in the lower right panel.

RevErbα

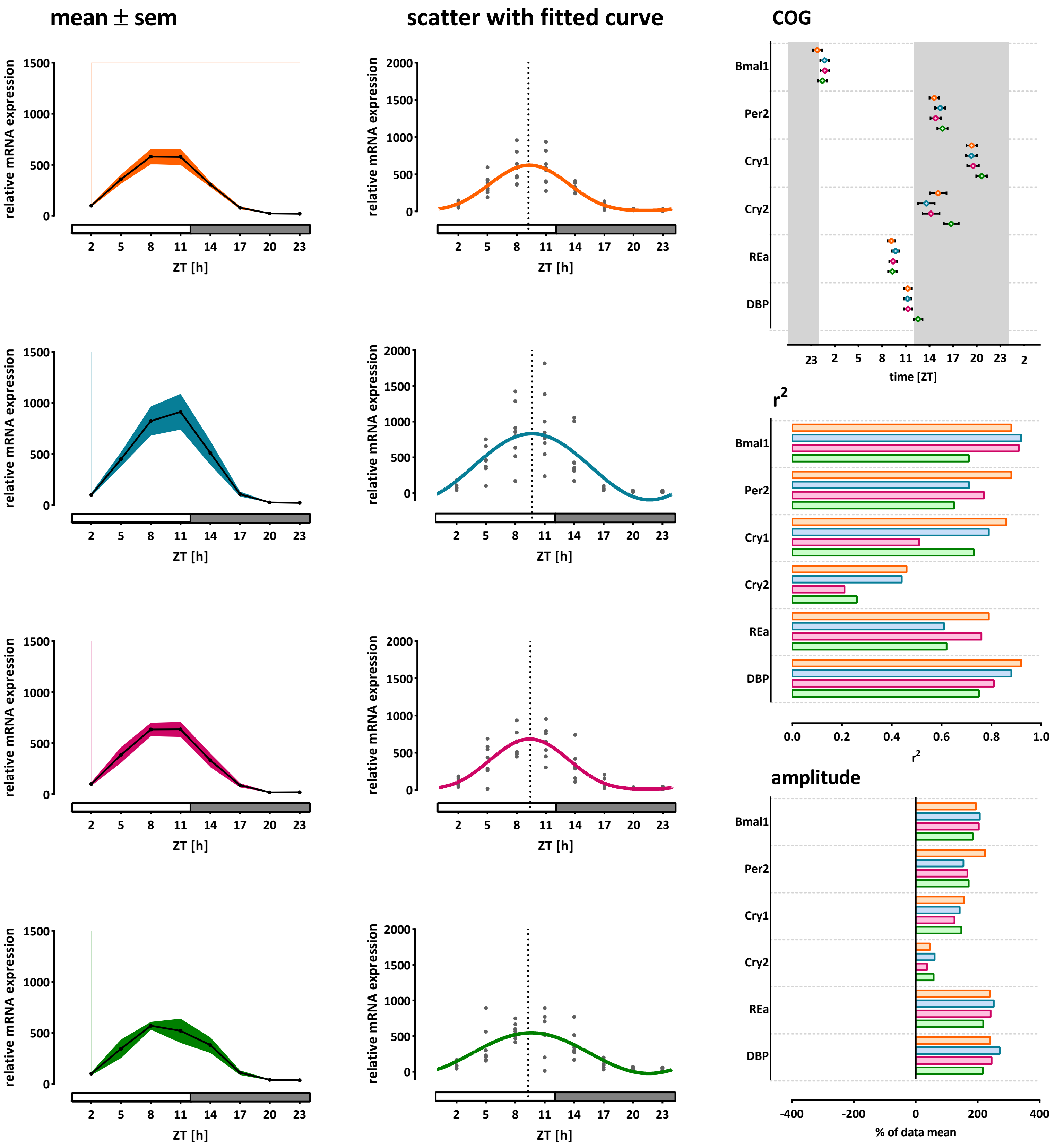

PCR data are shown in the left pannel (mean ± sem) and middle panel (scatter plot). All data are corrected for housekeeping gene expression and are expressed relative to ZT2 to allow for comparison between different PCR plates.

Circwave fitted curves are shown in the panel in the middle.
COG(Centre Of Gravity) is a general phase marker. Depicted in the upper right panel and by the dotted line in the scatterplots in the middle panel.
r^2 describes goodness of fit, shown in the right hand panel. 1.0 indicates perfect description of individual data points and thus little variation between animals.
amplitudes are expressed as max-min/data mean, shown in the lower right panel.

DBP

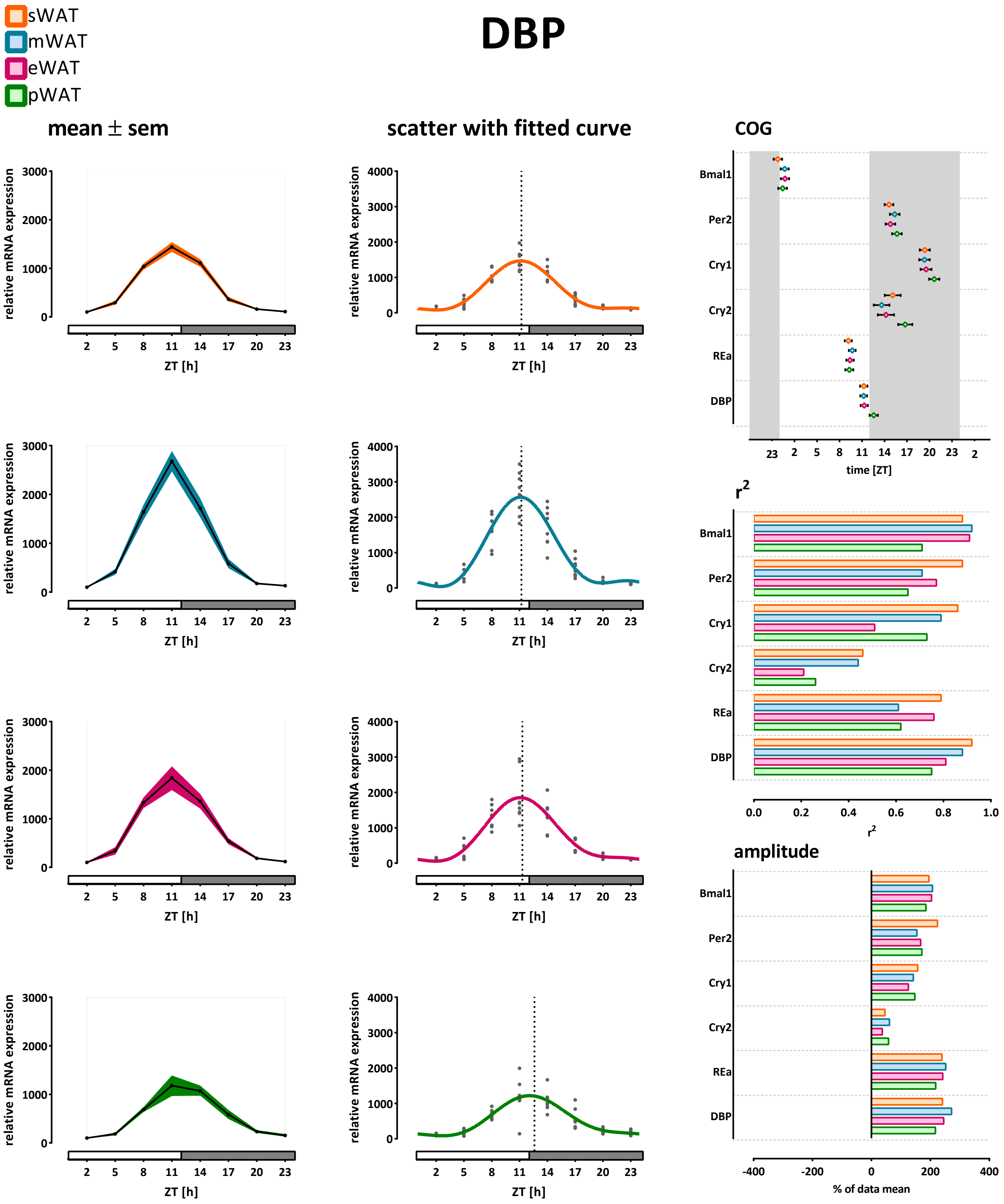

PCR data are shown in the left pannel (mean ± sem) and middle panel (scatter plot). All data are corrected for housekeeping gene expression and are expressed relative to ZT2 to allow for comparison between different PCR plates.

Circwave fitted curves are shown in the panel in the middle.
COG(Centre Of Gravity) is a general phase marker. Depicted in the upper right panel and by the dotted line in the scatterplots in the middle panel.
r^2 describes goodness of fit, shown in the right hand panel. 1.0 indicates perfect description of individual data points and thus little variation between animals.
amplitudes are expressed as max-min/data mean, shown in the lower right panel.

SREBP1c

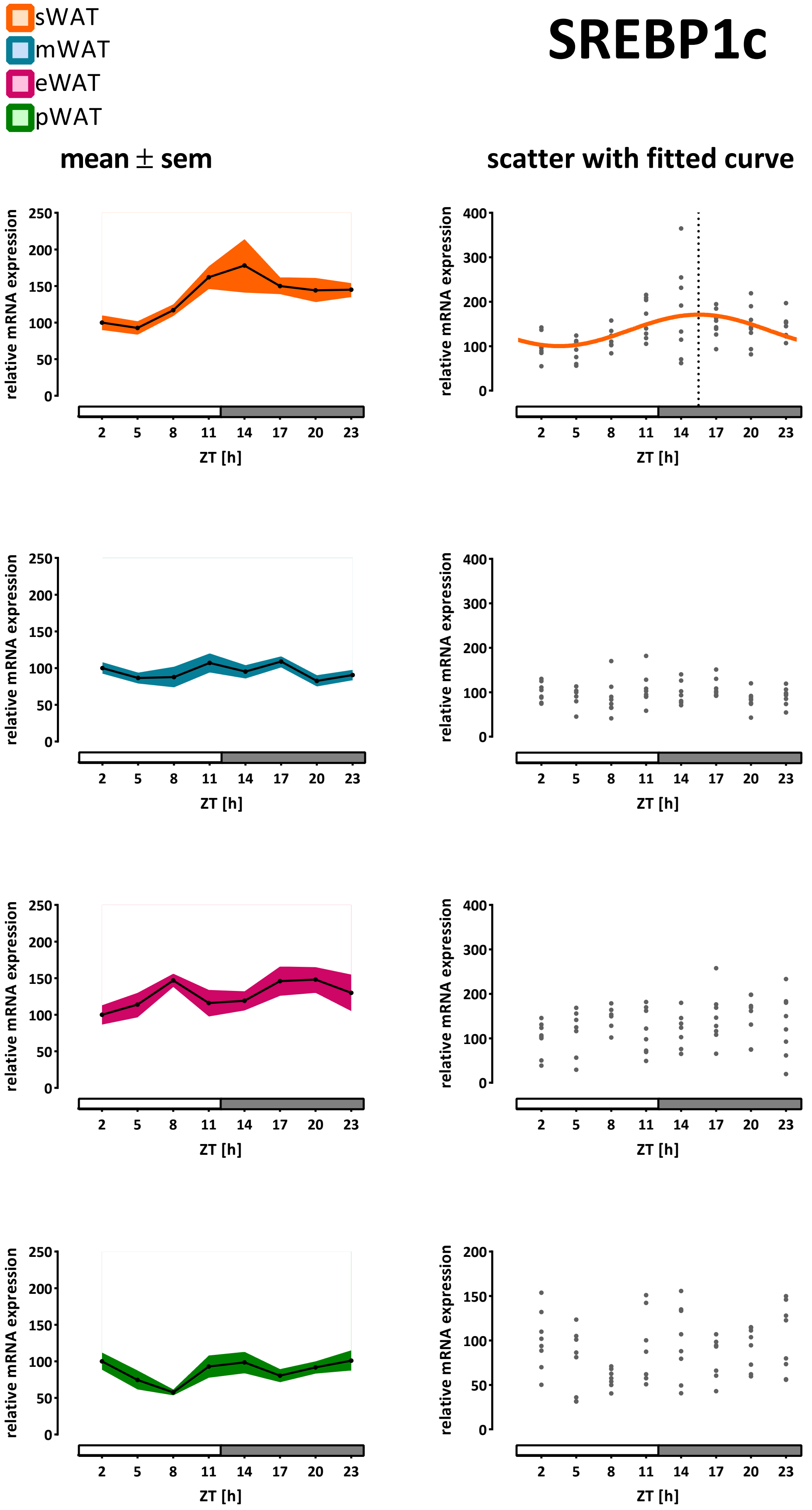

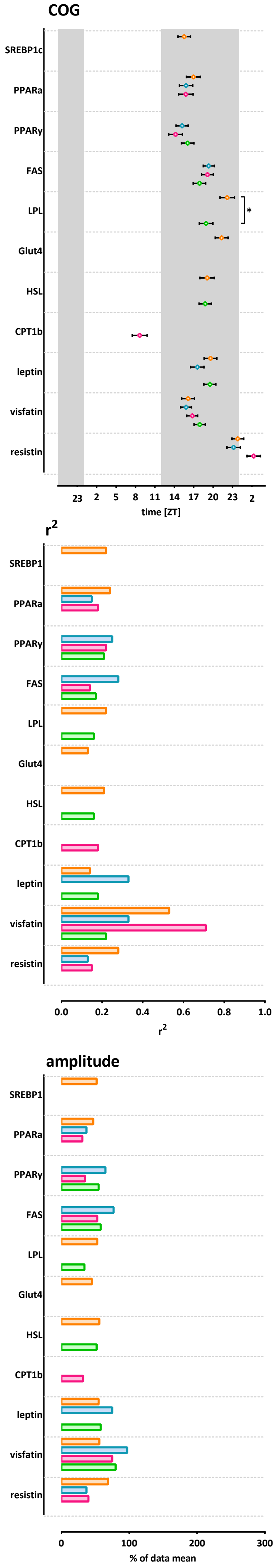

PCR data are shown in the left pannel (mean ± sem) and middle panel (scatter plot). All data are corrected for housekeeping gene expression and are expressed relative to ZT2 to allow for comparison between different PCR plates.

Circwave fitted curves are shown in the panel in the middle.
COG(Centre Of Gravity) is a general phase marker. Depicted in the upper right panel and by the dotted line in the scatterplots in the middle panel.
r^2 describes goodness of fit, shown in the right hand panel. 1.0 indicates perfect description of individual data points and thus little variation between animals.
amplitudes are expressed as max-min/data mean, shown in the lower right panel.

PPARα

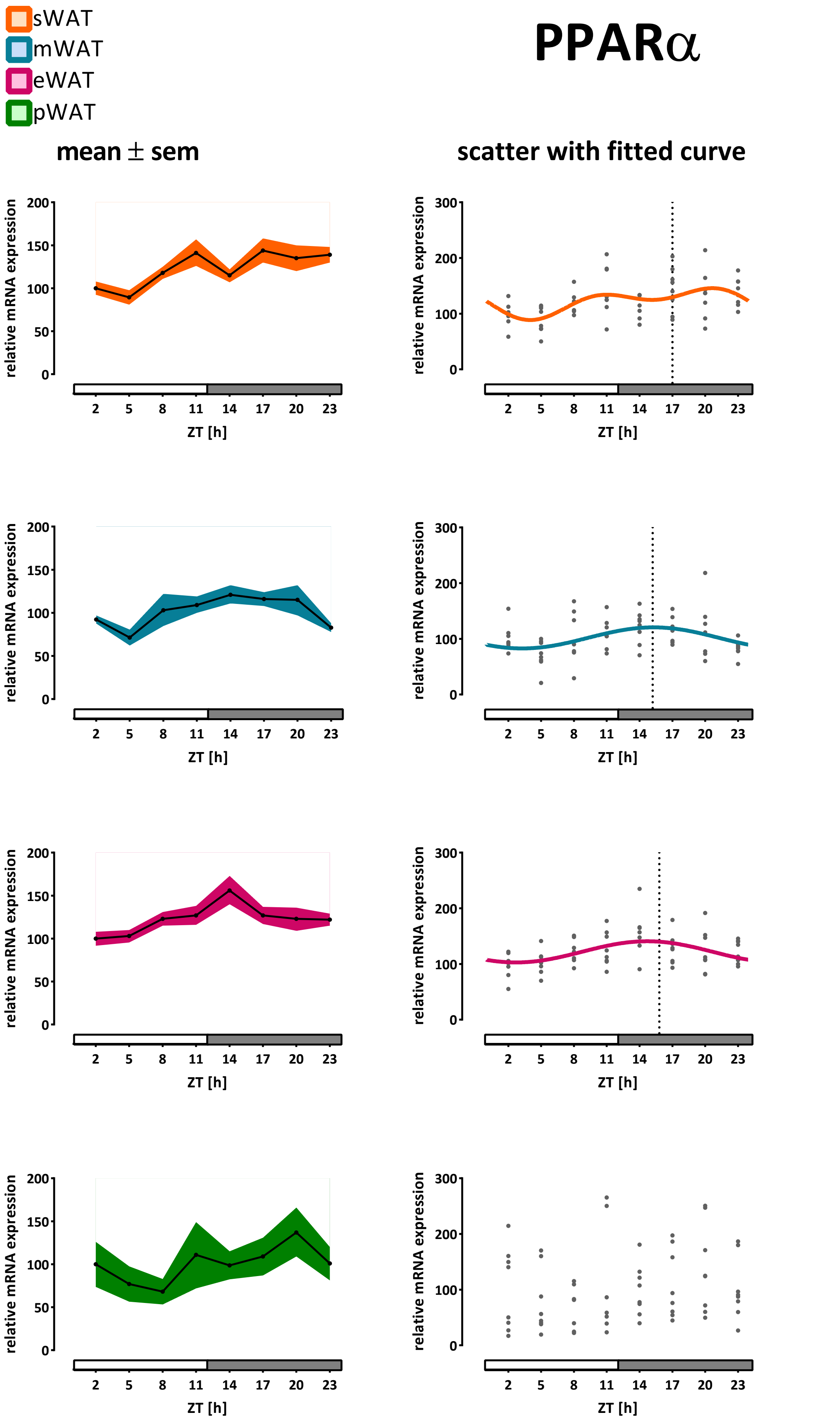

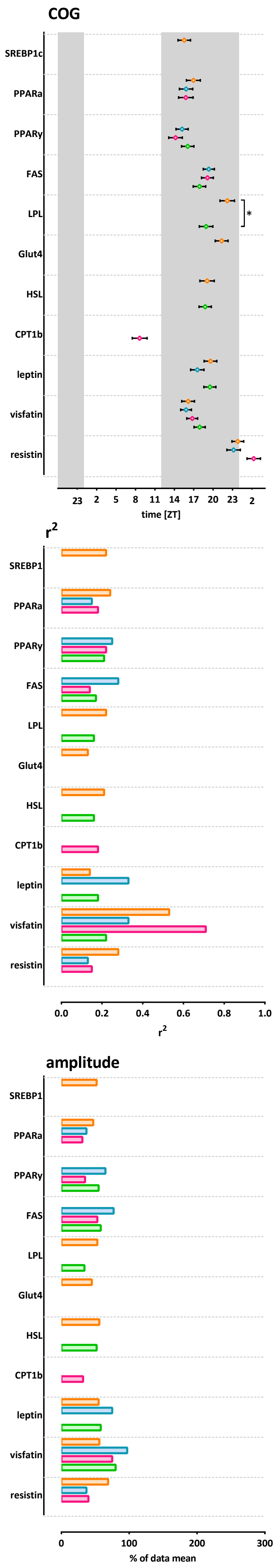

PCR data are shown in the left pannel (mean ± sem) and middle panel (scatter plot). All data are corrected for housekeeping gene expression and are expressed relative to ZT2 to allow for comparison between different PCR plates.

Circwave fitted curves are shown in the panel in the middle.
COG(Centre Of Gravity) is a general phase marker. Depicted in the upper right panel and by the dotted line in the scatterplots in the middle panel.
r^2 describes goodness of fit, shown in the right hand panel. 1.0 indicates perfect description of individual data points and thus little variation between animals.
amplitudes are expressed as max-min/data mean, shown in the lower right panel.

PPARγ

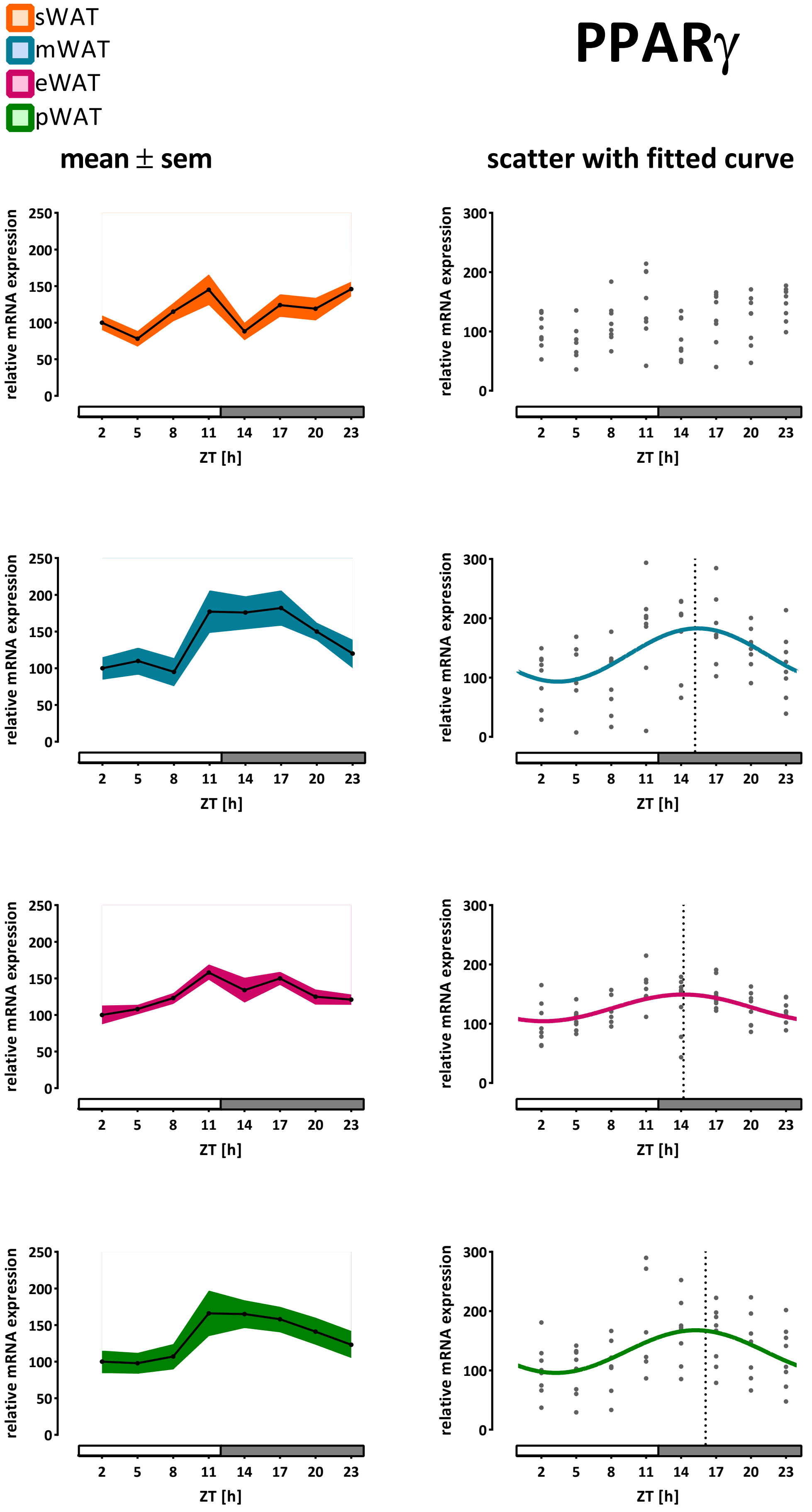

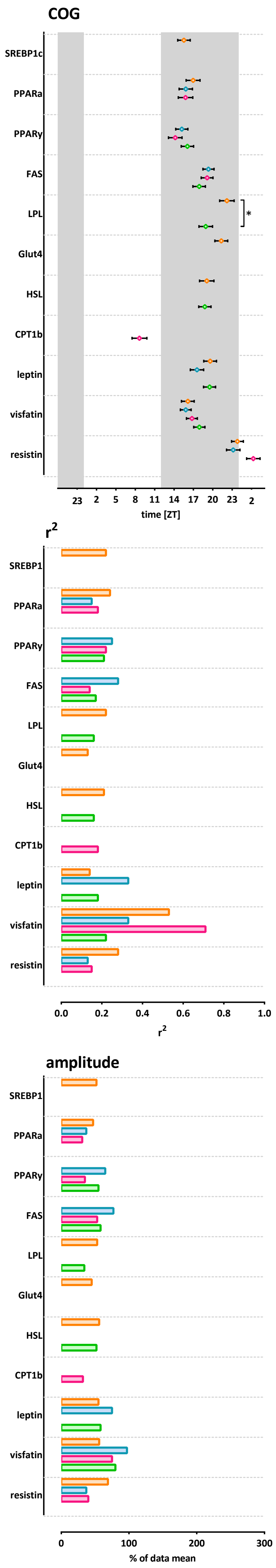

PCR data are shown in the left pannel (mean ± sem) and middle panel (scatter plot). All data are corrected for housekeeping gene expression and are expressed relative to ZT2 to allow for comparison between different PCR plates.

Circwave fitted curves are shown in the panel in the middle.
COG(Centre Of Gravity) is a general phase marker. Depicted in the upper right panel and by the dotted line in the scatterplots in the middle panel.
r^2 describes goodness of fit, shown in the right hand panel. 1.0 indicates perfect description of individual data points and thus little variation between animals.
amplitudes are expressed as max-min/data mean, shown in the lower right panel.

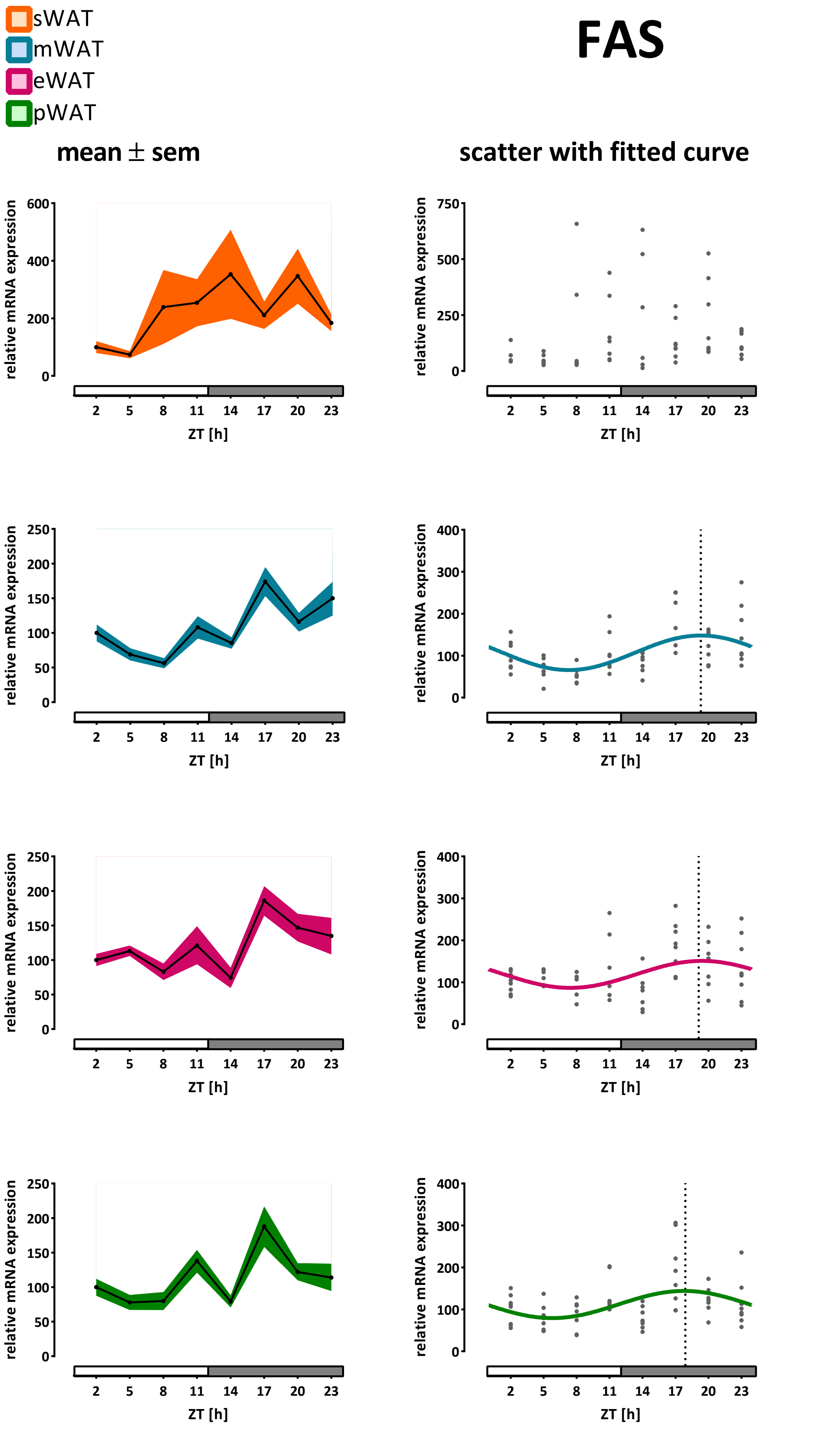

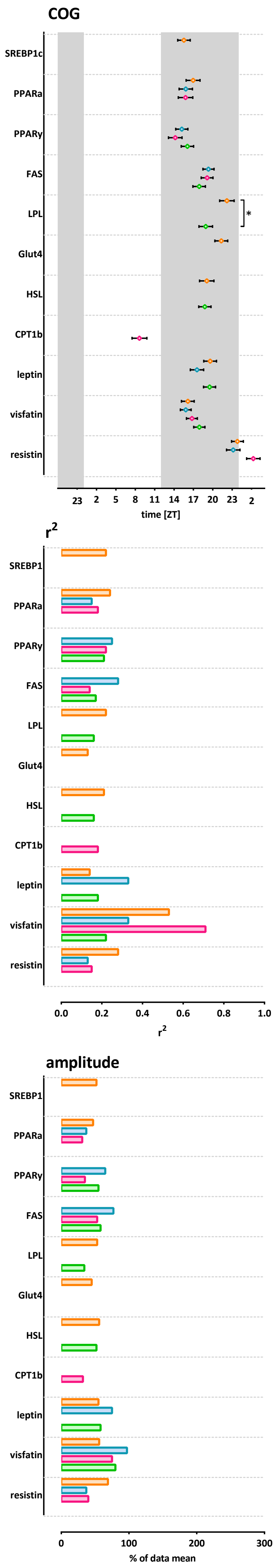

PCR data are shown in the left pannel (mean ± sem) and middle panel (scatter plot). All data are corrected for housekeeping gene expression and are expressed relative to ZT2 to allow for comparison between different PCR plates.

Circwave fitted curves are shown in the panel in the middle.
COG(Centre Of Gravity) is a general phase marker. Depicted in the upper right panel and by the dotted line in the scatterplots in the middle panel.
***r*^{2}** describes goodness of fit, shown in the right hand panel. 1.0 indicates perfect description of individual data points and thus little variation between animals.
amplitudes are expressed as max-min/data mean, shown in the lower right panel.

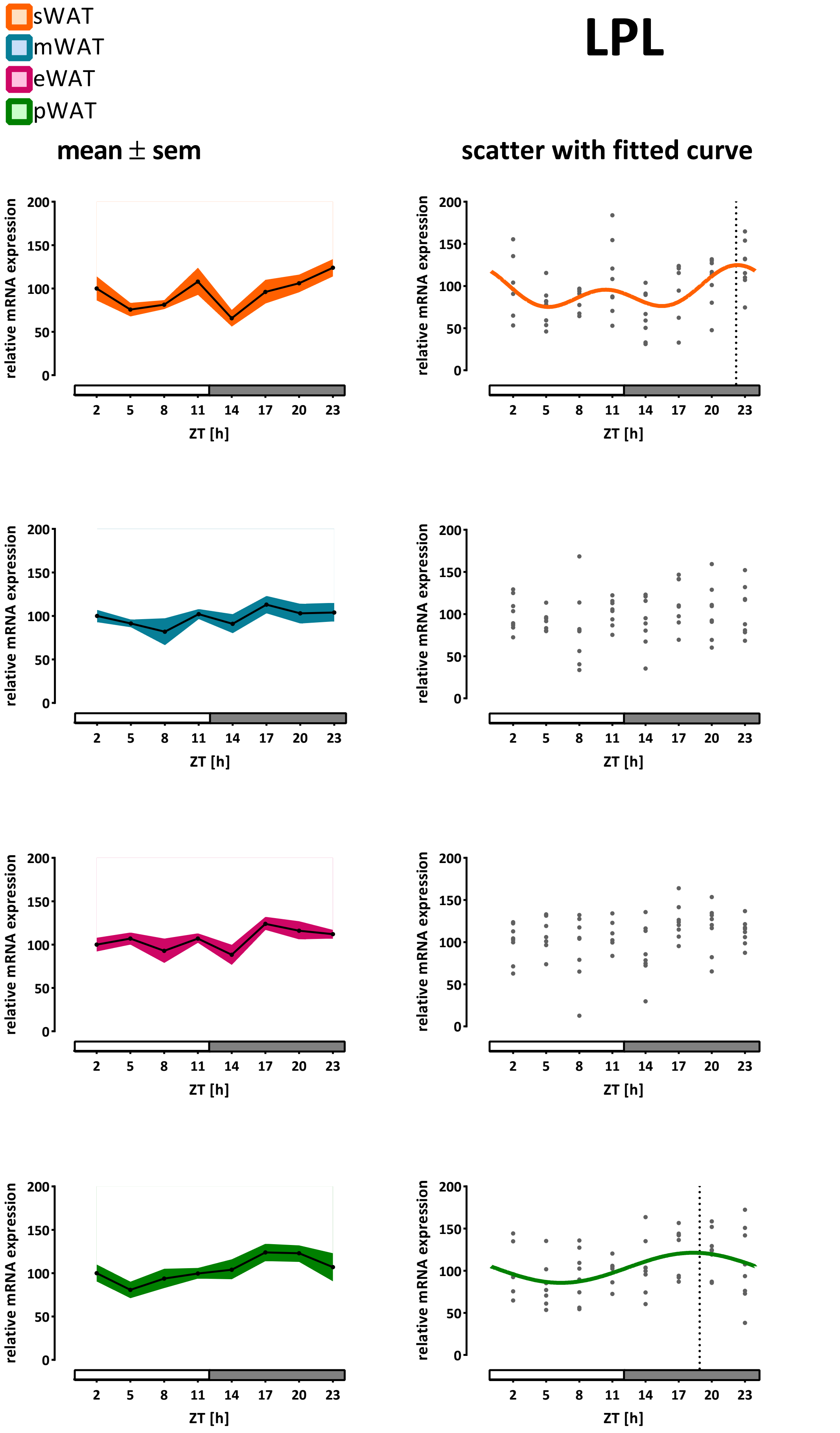

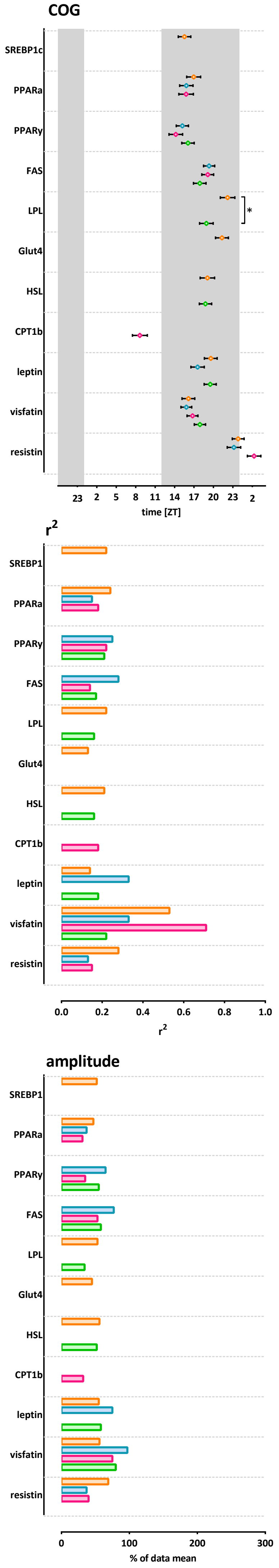

PCR data are shown in the left pannel (mean ± sem) and middle panel (scatter plot). All data are corrected for housekeeping gene expression and are expressed relative to ZT2 to allow for comparison between different PCR plates.

Circwave fitted curves are shown in the panel in the middle.
COG(Centre Of Gravity) is a general phase marker. Depicted in the upper right panel and by the dotted line in the scatterplots in the middle panel.
r^2 describes goodness of fit, shown in the right hand panel. 1.0 indicates perfect description of individual data points and thus little variation between animals.
amplitudes are expressed as max-min/data mean, shown in the lower right panel.

Glut4

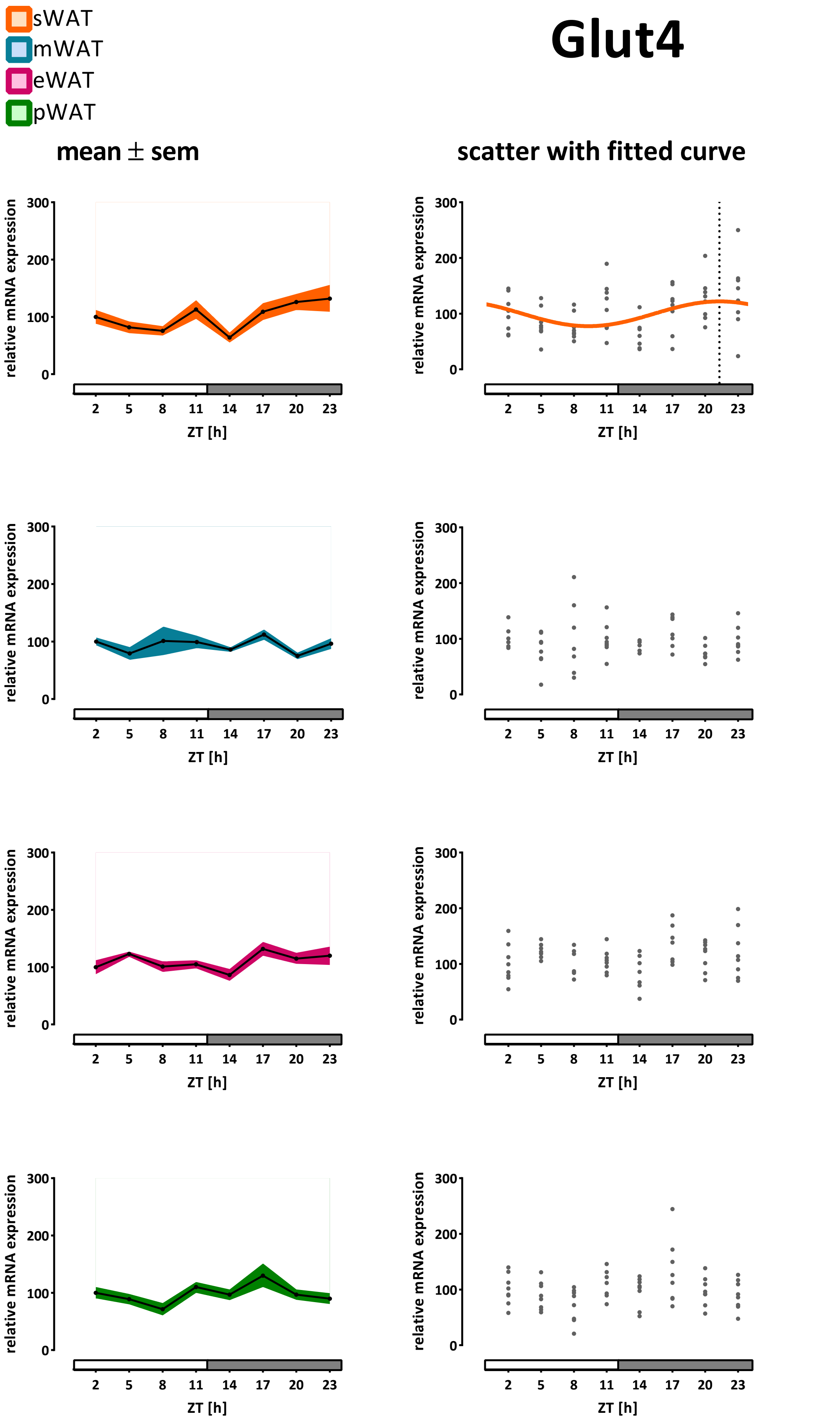

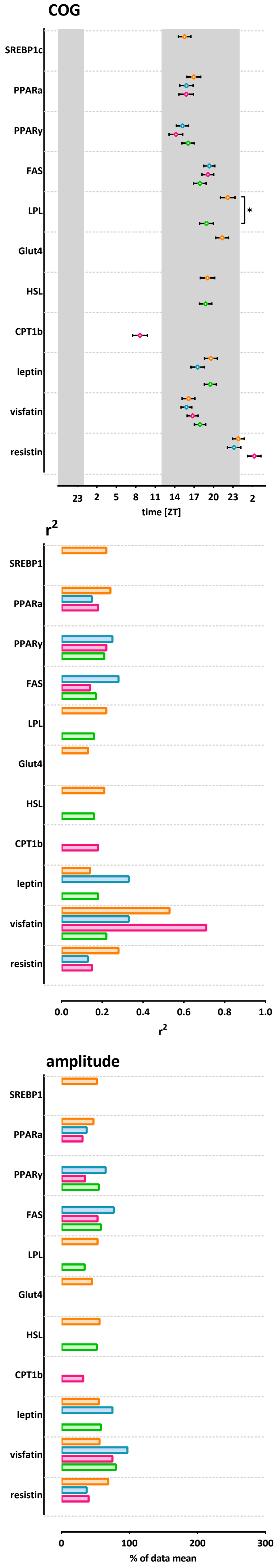

PCR data are shown in the left pannel (mean ± sem) and middle panel (scatter plot). All data are corrected for housekeeping gene expression and are expressed relative to ZT2 to allow for comparison between different PCR plates.

Circwave fitted curves are shown in the panel in the middle.
COG(Centre Of Gravity) is a general phase marker. Depicted in the upper right panel and by the dotted line in the scatterplots in the middle panel.
r² describes goodness of fit, shown in the right hand panel. 1.0 indicates perfect description of individual data points and thus little variation between animals.
amplitudes are expressed as max-min/data mean, shown in the lower right panel.

HSL

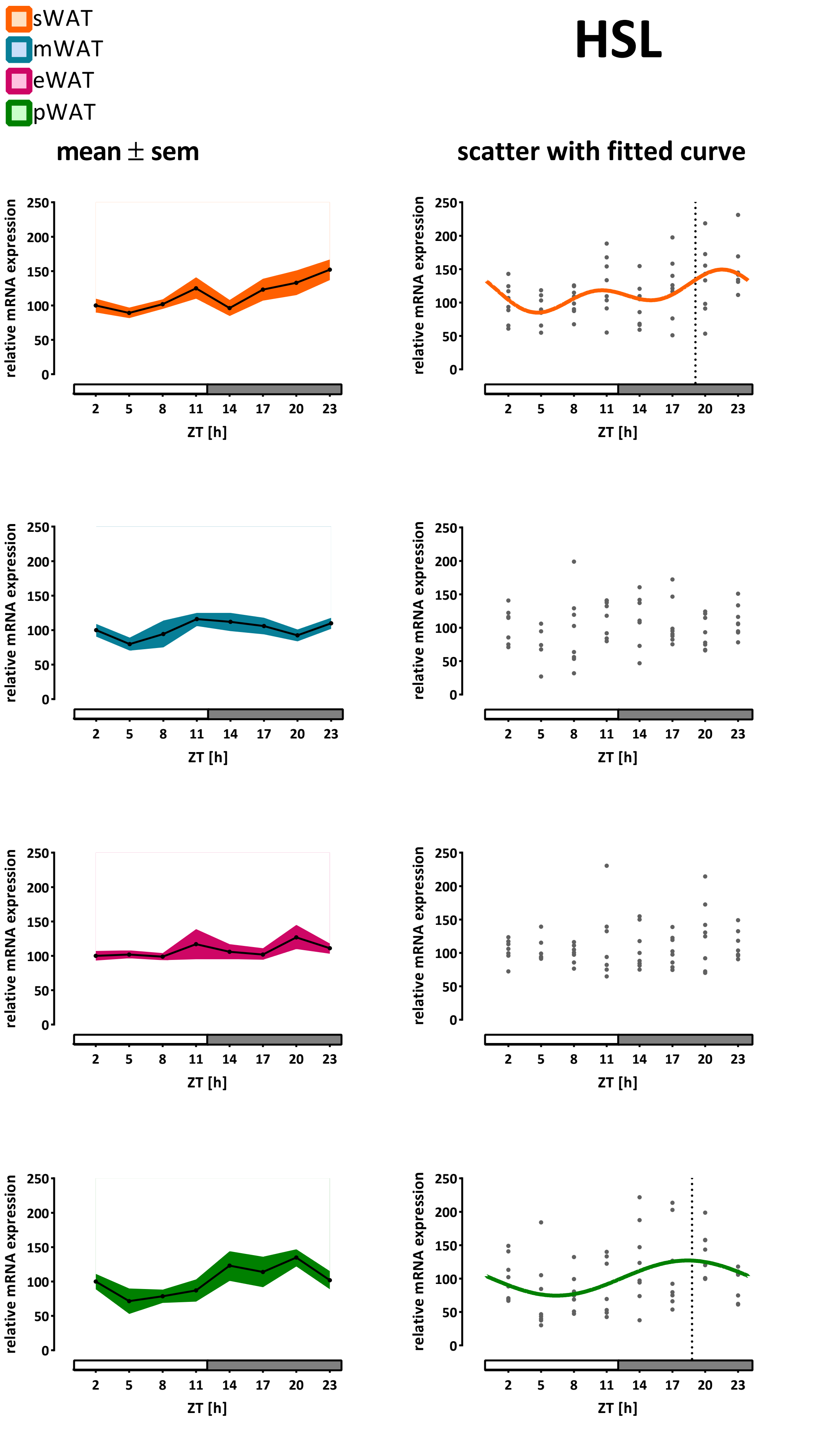

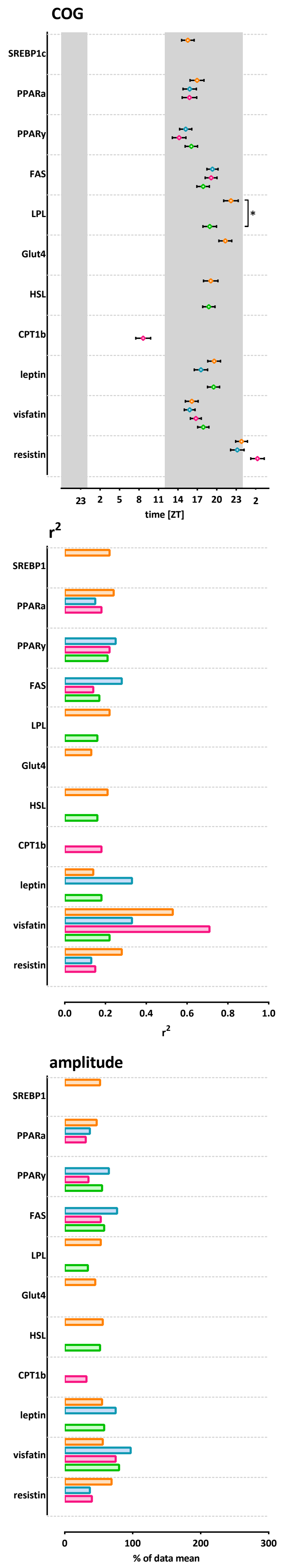

PCR data are shown in the left pannel (mean ± sem) and middle panel (scatter plot). All data are corrected for housekeeping gene expression and are expressed relative to ZT2 to allow for comparison between different PCR plates.

Circwave fitted curves are shown in the panel in the middle.
COG(Centre Of Gravity) is a general phase marker. Depicted in the upper right panel and by the dotted line in the scatterplots in the middle panel.
r^2 describes goodness of fit, shown in the right hand panel. 1.0 indicates perfect description of individual data points and thus little variation between animals.
amplitudes are expressed as max-min/data mean, shown in the lower right panel.

CPT1b

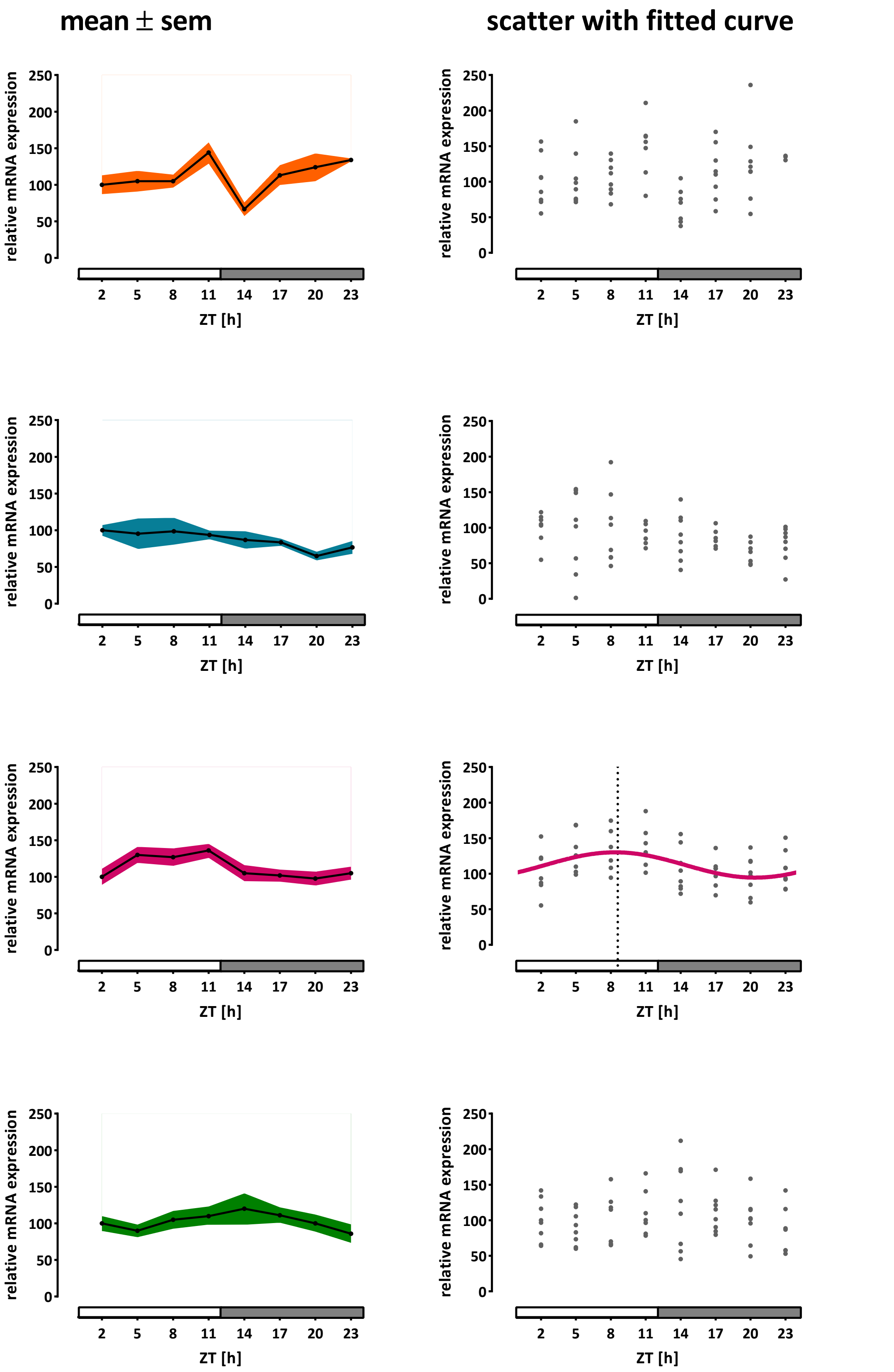

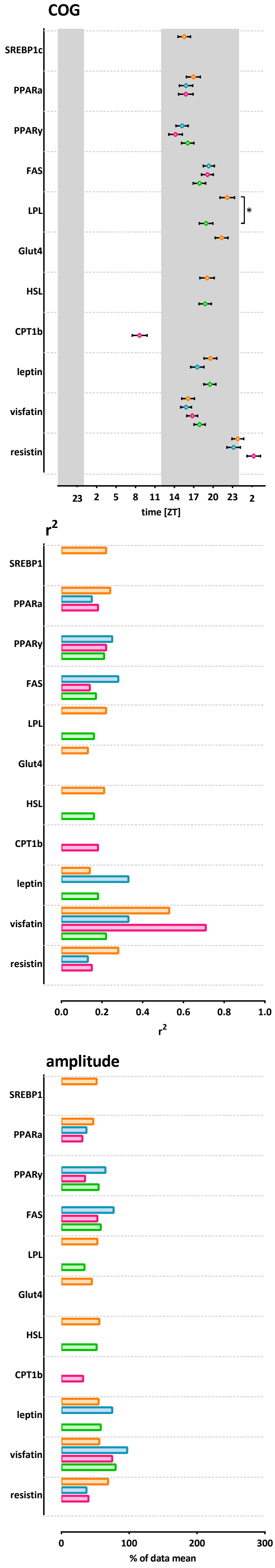

PCR data are shown in the left pannel (mean ± sem) and middle panel (scatter plot). All data are corrected for housekeeping gene expression and are expressed relative to ZT2 to allow for comparison between different PCR plates.

Circwave fitted curves are shown in the panel in the middle.
COG(Centre Of Gravity) is a general phase marker. Depicted in the upper right panel and by the dotted line in the scatterplots in the middle panel.
r^2 describes goodness of fit, shown in the right hand panel. 1.0 indicates perfect description of individual data points and thus little variation between animals.
amplitudes are expressed as max-min/data mean, shown in the lower right panel.

leptin

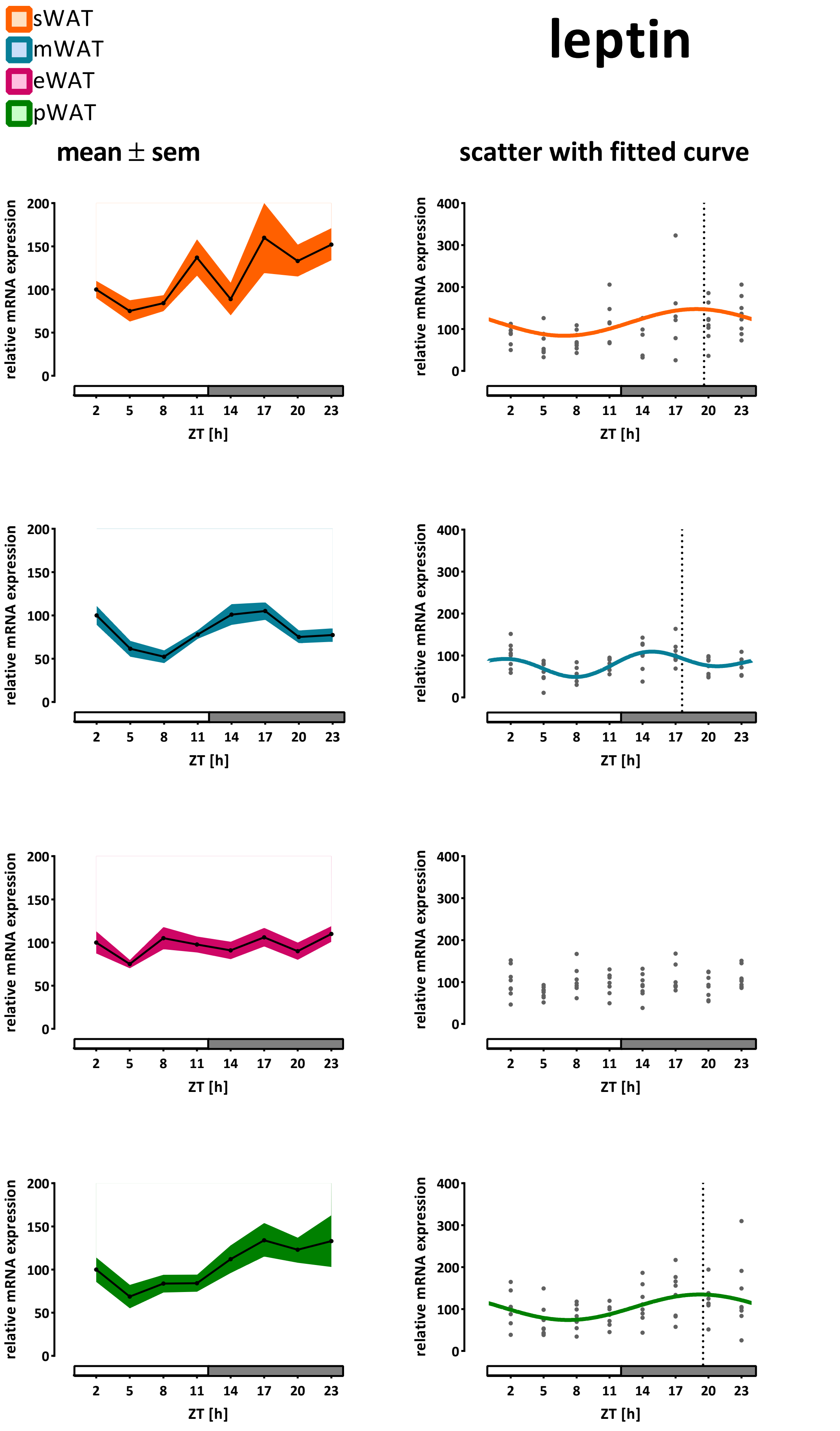

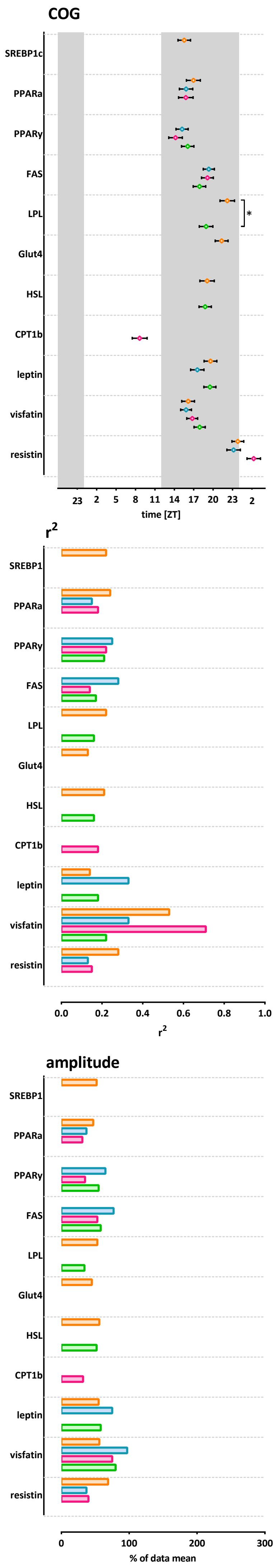

PCR data are shown in the left pannel (mean ± sem) and middle panel (scatter plot). All data are corrected for housekeeping gene expression and are expressed relative to ZT2 to allow for comparison between different PCR plates.

Circwave fitted curves are shown in the panel in the middle.
COG(Centre Of Gravity) is a general phase marker. Depicted in the upper right panel and by the dotted line in the scatterplots in the middle panel.
r^2 describes goodness of fit, shown in the right hand panel. 1.0 indicates perfect description of individual data points and thus little variation between animals.
amplitudes are expressed as max-min/data mean, shown in the lower right panel.

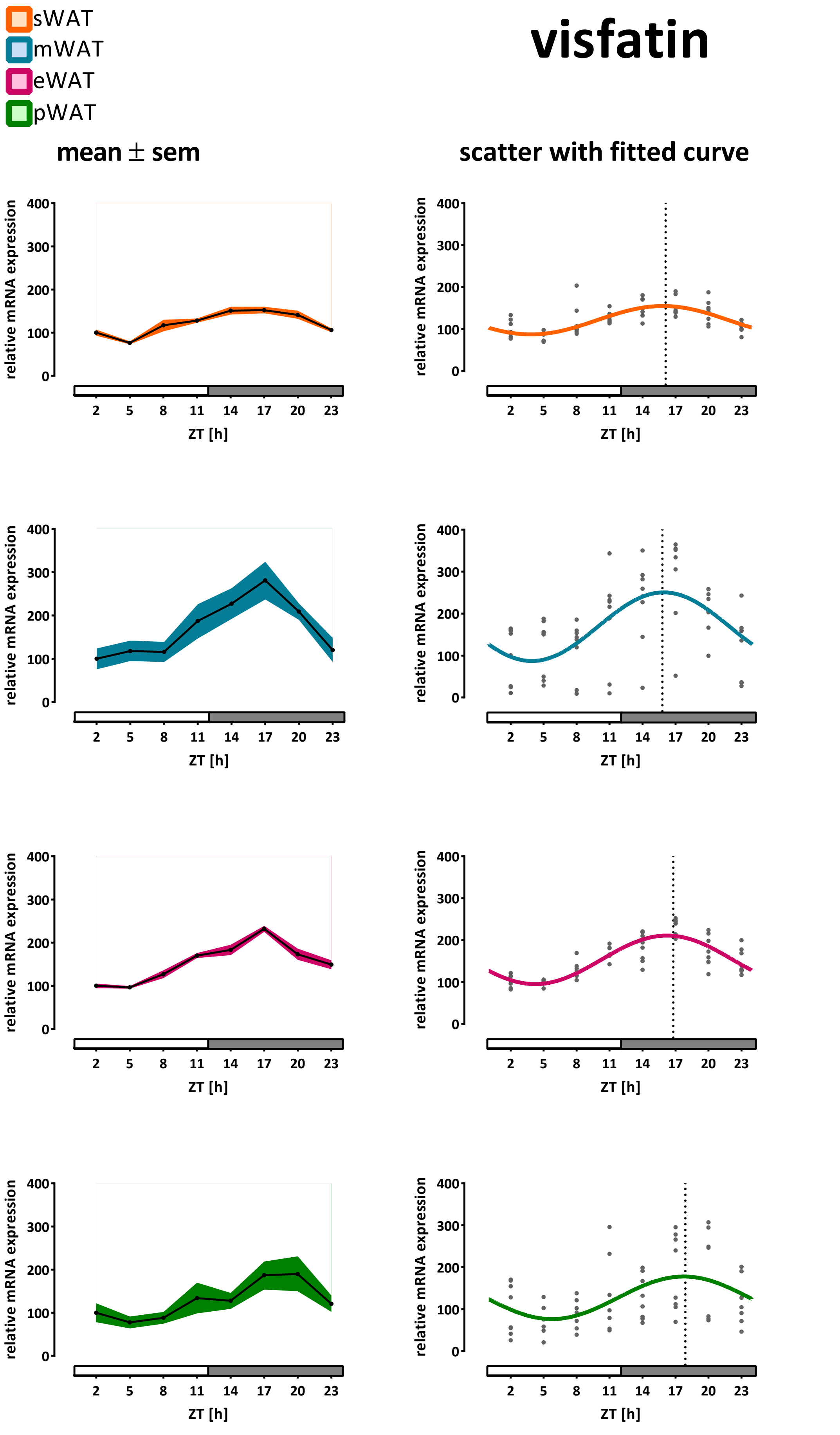

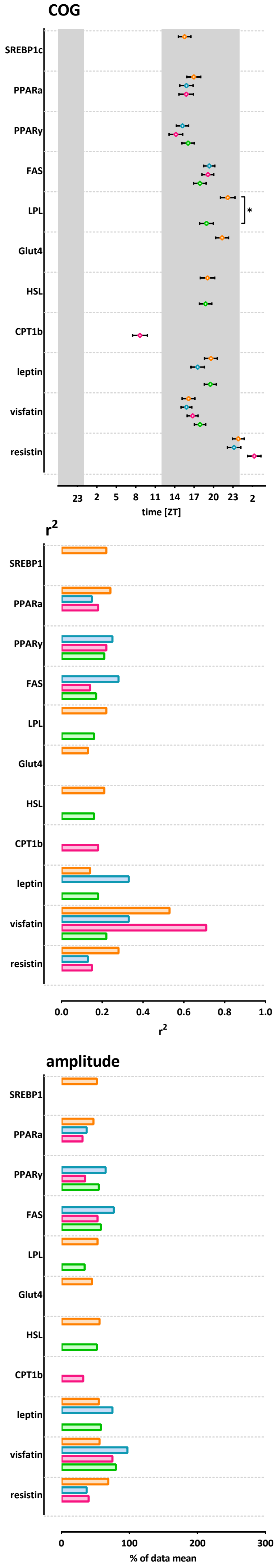

PCR data are shown in the left pannel (mean ± sem) and middle panel (scatter plot). All data are corrected for housekeeping gene expression and are expressed relative to ZT2 to allow for comparison between different PCR plates.

Circwave fitted curves are shown in the panel in the middle.
COG(Centre Of Gravity) is a general phase marker. Depicted in the upper right panel and by the dotted line in the scatterplots in the middle panel.
r² describes goodness of fit, shown in the right hand panel. 1.0 indicates perfect description of individual data points and thus little variation between animals.
amplitudes are expressed as max-min/data mean, shown in the lower right panel.

resistin

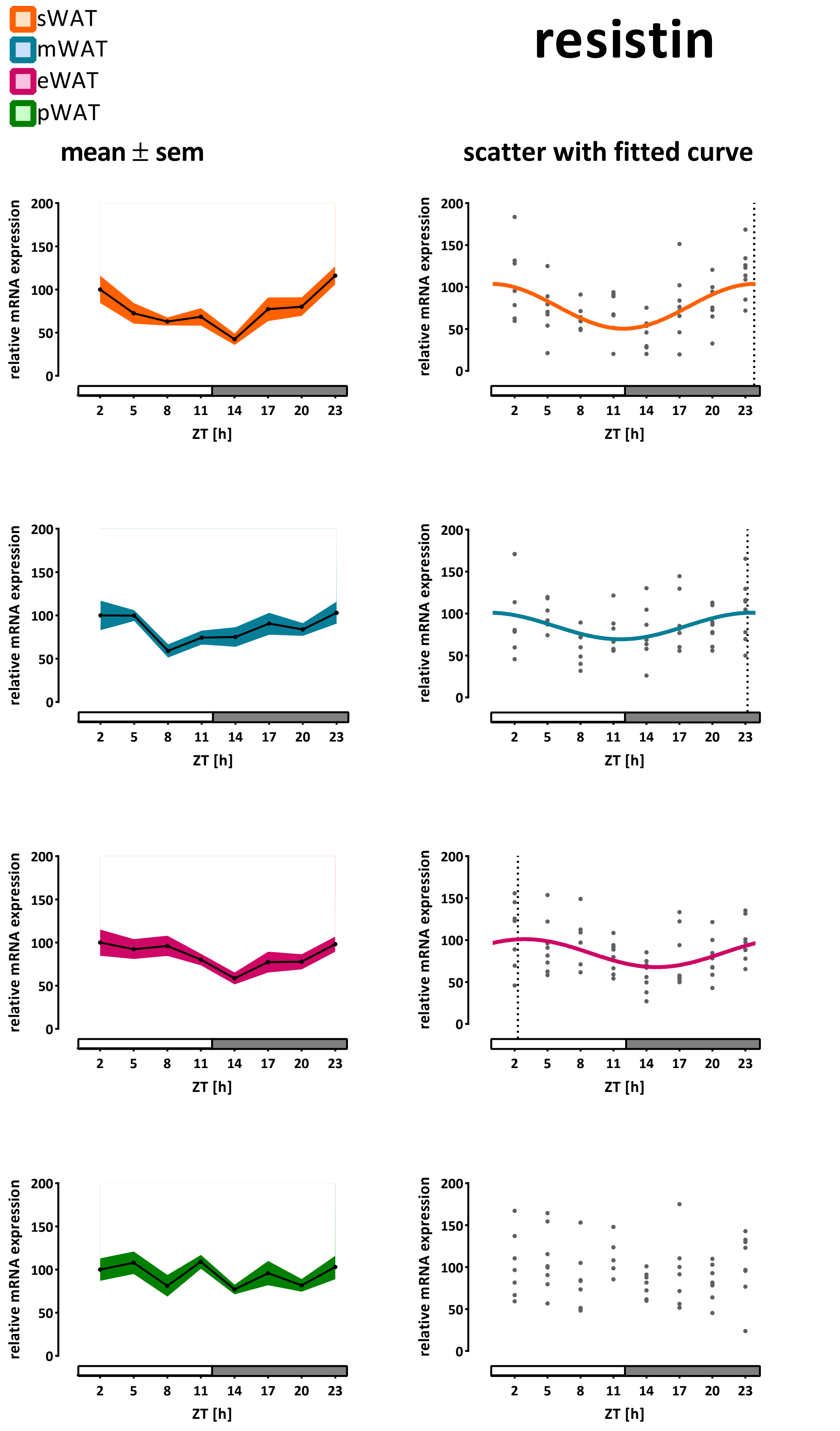

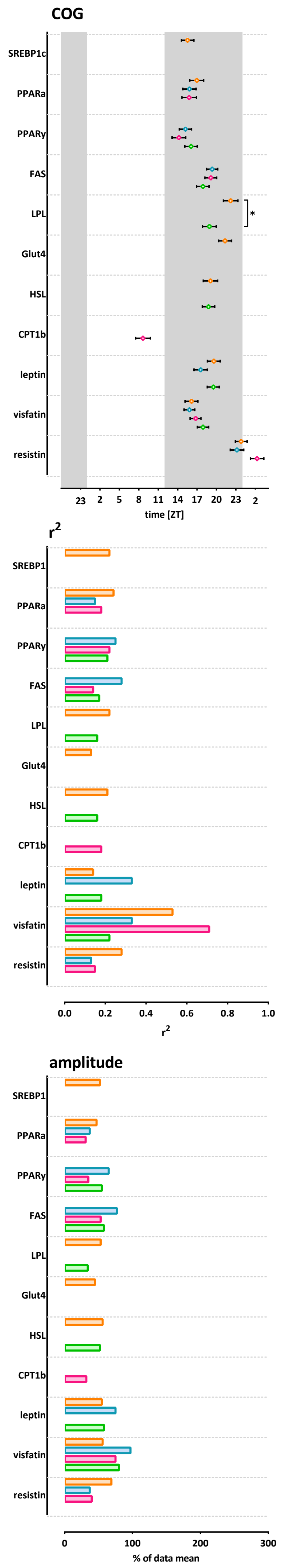

PCR data are shown in the left pannel (mean ± sem) and middle panel (scatter plot). All data are corrected for housekeeping gene expression and are expressed relative to ZT2 to allow for comparison between different PCR plates.

Circwave fitted curves are shown in the panel in the middle.
COG(Centre Of Gravity) is a general phase marker. Depicted in the upper right panel and by the dotted line in the scatterplots in the middle panel.
r² describes goodness of fit, shown in the right hand panel. 1.0 indicates perfect description of individual data points and thus little variation between animals.
amplitudes are expressed as max-min/data mean, shown in the lower right panel.
