# Supplementary material for: Daily Gene Expression Rhythms in Rat White Adipose Tissue Do Not Differ Between Subcutaneous and Intra-Abdominal Depots
Source: Front Endocrinol (Lausanne). 2018 Apr 30;9:206. doi: 10.3389/fendo.2018.00206 (PMC5936761; doi:10.3389/fendo.2018.00206)

	pWAT	eWAT	mWAT	sWAT
Number of XY Pairs	60	60	60	58
Pearson r	0.3049	0.1352	0.5883	0.2833
95% confidence interval	0.05524 to 0.5188	-0.1230 to 0.3763	0.3930 to 0.7328	0.02693 to 0.5047
P value (two-tailed)	0.0178	0.3029	< 0.0001	0.0312
P value summary	*	ns	***	*
Is the correlation significant? (alpha=0.05)	Yes	No	Yes	Yes
R square	0.09299	0.01829	0.3461	0.08027

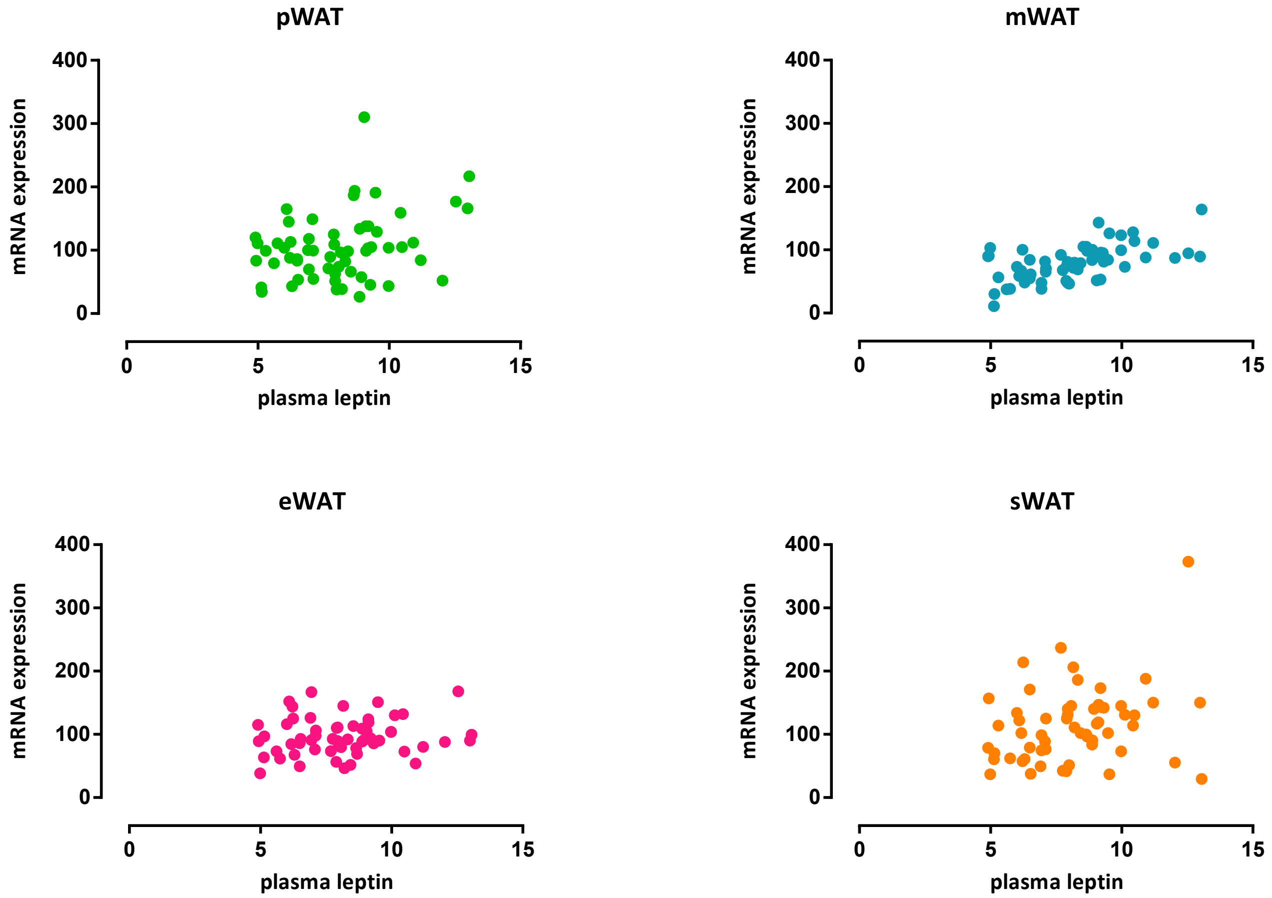

Supplement: Figure S2 — Correlation between plasma leptin concentrations and leptin mRNA expression in mesenteric WAT. [file image_2.PDF]
