# Supplementary material for: Daily Gene Expression Rhythms in Rat White Adipose Tissue Do Not Differ Between Subcutaneous and Intra-Abdominal Depots
Source: Front Endocrinol (Lausanne). 2018 Apr 30;9:206. doi: 10.3389/fendo.2018.00206 (PMC5936761; doi:10.3389/fendo.2018.00206)
Supplement: Table S1 — The number of samples after exclusion of outliers for the PCR results at each time point and for each gene investigated. [file table_1.PDF]

Supplemental table 1 - PCR data n per group after exclusion of outliers

Gene	ZT	pWAT	sWAT	eWAT	mWAT
**Bmal1**	2	8	8	8	8
	5	8	8	8	7
	8	8	8	7	8
	11	6	7	8	7
	14	7	8	8	7
	17	8	8	8	8
	20	8	8	7	8
	23	8	8	8	8
**Per2**	2	8	8	8	8
	5	8	8	7	7
	8	8	8	8	8
	11	7	8	8	8
	14	8	8	8	8
	17	7	8	8	8
	20	7	8	7	8
	23	8	8	8	7
**Cry1**	2	8	8	8	8
	5	8	7	8	7
	8	8	8	8	7
	11	7	7	8	8
	14	7	8	8	8
	17	8	7	8	8
	20	8	8	8	8
	23	8	8	8	8
**Cry2**	2	8	8	8	8
	5	8	8	8	7
	8	8	8	7	8
	11	7	8	8	8
	14	8	8	8	8
	17	8	8	7	8
	20	8	8	8	8
	23	7	8	8	8
**Rea**	2	8	8	8	8
	5	8	8	8	8
	8	8	8	7	8
	11	7	8	8	8
	14	7	7	8	8
	17	7	8	8	8
	20	8	8	6	8
	23	7	8	8	8
**DBP**	2	8	8	8	8
	5	8	8	8	8
	8	7	8	8	8
	11	7	8	8	8
	14	8	8	8	8
	17	8	8	8	8
	20	7	8	7	8
	23	7	8	8	8
**SREBP1**	2	8	8	8	8
	5	8	8	8	8
	8	7	8	7	8
	11	7	8	8	8
	14	8	8	8	8
	17	7	8	8	8
	20	8	8	8	8
	23	8	8	8	8

**PPARa**	2	8	8	8	8
	5	8	8	8	8
	8	7	8	7	7
	11	7	8	8	8
	14	8	8	7	8
	17	8	8	8	8
	20	8	8	8	8
	23	8	8	8	8
**PPARy**	2	8	8	8	8
	5	8	8	8	8
	8	7	8	8	8
	11	7	8	8	8
	14	8	8	8	8
	17	8	8	8	7
	20	8	8	8	8
	23	8	8	8	8
**FAS**	2	8	7	8	8
	5	8	8	6	8
	8	8	8	7	7
	11	7	8	8	8
	14	8	7	8	8
	17	8	8	8	8
	20	7	8	8	8
	23	8	8	8	8
**LPL**	2	8	7	8	8
	5	8	8	8	7
	8	8	8	8	8
	11	7	8	8	8
	14	8	8	8	8
	17	8	7	8	8
	20	8	8	8	8
	23	8	8	8	8
**GLUT4**	2	8	8	8	8
	5	8	8	8	8
	8	8	8	7	7
	11	7	8	8	8
	14	8	8	8	7
	17	8	8	8	8
	20	8	8	8	7
	23	8	8	8	8
**HSL**	2	8	8	8	8
	5	8	8	8	8
	8	8	8	7	8
	11	7	8	7	8
	14	8	8	8	8
	17	8	8	8	8
	20	8	8	8	8
	23	8	7	8	8
**CPT1b**	2	8	8	8	8
	5	8	8	7	8
	8	8	8	7	8
	11	7	8	8	7
	14	8	7	8	8
	17	8	8	7	7
	20	8	8	8	7
	23	7	3	8	8
**Leptin**	2	8	7	8	8
	5	8	8	8	8
	8	8	8	7	7

	11	7	7	8	8
	14	8	7	8	8
	17	8	7	8	8
	20	8	8	8	8
	23	8	8	8	7
**Visfatin**	2	8	8	8	8
	5	8	8	7	8
	8	7	8	7	8
	11	7	8	7	8
	14	8	7	8	8
	17	8	8	8	7
	20	7	8	8	8
	23	8	8	8	8
**Resistin**	2	8	8	8	8
	5	8	7	8	7
	8	8	8	7	7
	11	7	7	8	8
	14	8	8	8	8
	17	8	8	8	7
	20	8	7	8	8
	23	8	8	8	8
